# Transcriptional signature of induced neurons differentiates virologically suppressed people with HIV from people without HIV

**DOI:** 10.1172/jci.insight.190445

**Published:** 2025-11-25

**Authors:** Philipp N. Ostermann, Youjun Wu, Scott A. Bowler, Samuel Martínez-Meza, Mohammad Adnan Siddiqui, David H. Meyer, Alberto Herrera, Brandon A. Sealy, Mega Sidharta, Kiran Ramnarine, Leslie Ann St. Bernard, Desiree Byrd, R. Brad Jones, Masahiro Yamashita, Douglas F. Nixon, Lishomwa C. Ndhlovu, Ting Zhou, Teresa H. Evering

**Affiliations:** 1 Division of Infectious Diseases, Department of Medicine, Weill Cornell Medicine, New York, New York, USA.; 2 The SKI Stem Cell Research Facility, The Center for Stem Cell Biology and Developmental Biology Program, Sloan Kettering Institute, New York, New York, USA.; 3 Institute of Translational Research, Feinstein Institutes for Medical Research, Northwell Health, Manhasset, New York, USA.; 4 Aaron Diamond AIDS Research Center, Columbia University Vagelos College of Physicians and Surgeons, New York, New York, USA.; 5 Institute for Genome Stability in Aging and Disease, University Hospital and University of Cologne, Cologne, Germany.; 6 Cologne Excellence Cluster for Cellular Stress Responses in Aging-Associated Diseases (CECAD), Center for Molecular Medicine Cologne (CMMC), University of Cologne, Cologne, Germany.; 7 Department of Neurology, The Icahn School of Medicine at Mount Sinai, New York, New York, USA.; 8 Department of Psychology, Queens College and the Graduate Center, City University of New York, New York, New York, USA.

**Keywords:** AIDS/HIV, Neuroscience, Neurological disorders

## Abstract

Neurocognitive impairment is a prevalent comorbidity in virologically suppressed people living with HIV (PLWH), yet the underlying mechanisms remain elusive and treatments lacking. We explored use of participant-derived directly induced neurons (iNs) to model neuronal biology and injury in PLWH. iNs retain age- and disease-related donor features, providing unique opportunities to reveal important aspects of neurological disorders. We obtained primary dermal fibroblasts from 6 virologically suppressed PLWH and 7 matched people without HIV (PWOH). iNs were generated using transcription factors NGN2 and ASCL1 and validated by immunocytochemistry, single-cell RNA-Seq, and electrophysiological recordings. Transcriptomic aging analyses confirmed retention of donor age-related signatures. Bulk RNA-Seq identified 29 significantly differentially expressed genes between PLWH and PWOH iNs. Of these, 16 were downregulated and 13 upregulated in PLWH iNs. Protein-protein interaction network mapping indicated iNs from PLWH exhibited differences in extracellular matrix organization and synaptic transmission. *IFI27* was upregulated in PLWH iNs, complementing independent postmortem studies demonstrating elevated *IFI27* expression in PLWH-derived brain tissue. *FOXL2NB-FOXL2-LINC01391* expression was reduced in PLWH iNs and negatively correlated with neurocognitive impairment. Thus, we identified an iN gene signature of HIV revealing mechanisms of neurocognitive impairment in PLWH.

## Abstract 2

Direct reprogramming of skin fibroblasts into neurons reveals unique gene signatures indicative of HIV infection in the context of viral suppression.

## Introduction

Neurocognitive impairment (NCI) remains an important comorbidity in virologically suppressed people living with HIV (PLWH). Combined antiretroviral therapy (cART) has reduced the prevalence of the most severe forms, including HIV-associated dementia. However, milder forms of cognitive impairment remain prominent, with substantial burden and adverse consequences for daily living activities (1, 2). A 2020 meta-analysis determined the prevalence of HIV-1–related NCI to be 43.9% (3).

The cellular mechanisms underlying NCI in virologically suppressed PLWH are not well understood but are likely multifactorial. HIV-1 enters the brain as early as 2 weeks after infection, infecting multiple cell types, including T cells, microglia, brain-resident macrophages, and astrocytes (4–7). While neurons are not directly infected, the resulting neurotoxic environment impairs neuronal function, driving NCI in PLWH (7).

Transcriptomic analysis of postmortem brain samples from PLWH demonstrates that HIV-1 infection is associated with a differential neural gene expression across multiple pathways (e.g., axon guidance, endocytosis, synaptic transmission) associated with cognitive decline (8). However, most of these studies lacked non–HIV-1 controls (i.e., brain tissue from people without HIV [PWOH]), hampering understanding of direct HIV-1–associated differential neuronal gene expression (9). Moreover, analyzed gene expression may have been affected by comorbidities, antemortem living situations, and postmortem sample preparation.

To overcome these issues, differential gene expression has been analyzed in neurons from transgenic HIV-1 gp120-expressing mice, which reconstitute certain aspects of human HIV-1–induced neuropathology (10–12). However, HIV-1 does not naturally infect rodents, NCI in PLWH is influenced by factors beyond gp120, and mouse neuronal biology differs from that of humans. Thus, the need remains for a neuronal cell system reflecting the multifactorial nature of HIV-1 infection that allows transcriptional and functional analyses of neurons from virologically suppressed PLWH.

Protocols to generate induced neurons (iNs) by transdifferentiation of participant-derived fibroblasts enable capture of disease- and age-related neuronal features in vitro (13–16), recapitulating known aspects of neurodegenerative diseases and revealing previously unrecognized disease pathomechanisms in cell culture (13–15). Importantly, inducing the pluripotent stem cell state prior to neuronal differentiation, as is necessary for induced pluripotent stem cell–derived (iPSC-derived) neurons, erases most age- and disease-related characteristics, unlike the iN protocol (17). This is particularly important for studying diseases in which age is implicated in the pathogenesis, like Alzheimer’s disease (AD) or HIV-1–related NCI (14, 18).

Using a published iNs protocol that retains donor-specific disease- and age-related characteristics in vitro (14, 17, 19), we investigated whether iNs from virologically suppressed PLWH show differential gene expression compared with iNs from demographically matched PWOH.

## Results

### Generation of participant-derived induced neurons from people living with or without HIV.

**Figure 1:**
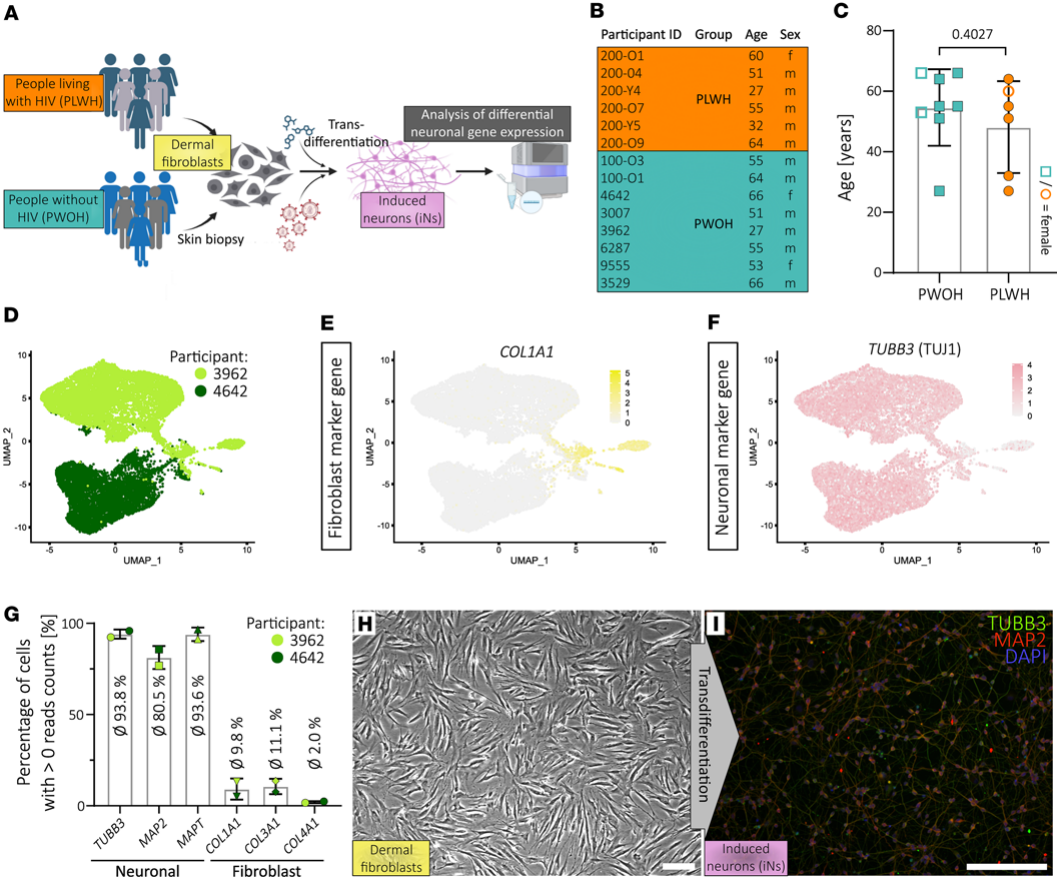
Transdifferentiation of skin fibroblasts derived from people living with HIV and controls generates induced neurons. (**A**) Scheme illustrating study outline. (**B**) Important participant information divided into people living with HIV-1 (PLWH) and without HIV-1 (PWOH). (**C**) Age distribution of the 2 groups of the study cohort. Statistical significance tested with unpaired, 2-tailed *t* test. Data presented as individual data points with mean ± SD and *P* value. (**D**) UMAP plot showing the sample origin of each data point during scRNA analysis. (**E** and **F**) Gene expression patterns of fibroblast marker gene *COL1A1* (**E**) and neuronal marker gene *TUBB3* (**F**). (**G**) Percentage of cells from scRNA analysis depicted in **D**–**F** that express the annotated neuronal and fibroblast marker genes. Data presented as individual data points with mean ± SD. (**H**) Microscopic image of dermal fibroblasts before transdifferentiation. (**I**) Microscopic image after immunocytochemistry of induced neurons (iNs) 3 days after FACS and stained for TUBB3 (TUJ1), MAP2, and nuclei (DAPI). (**H** and **I**) Scale bars are 20 μm and single-channel images are provided in Supplemental Figure 1F.

We generated iNs from 6 clinically well-characterized people chronically infected with HIV-1 and virologically suppressed on cART (HIV RNA < 50 copies/mL) and from 7 age- and sex-matched PWOH as controls (8 PWOH fibroblast samples were attempted; 1 did not yield sufficient iNs for analysis) following a published protocol (Figure 1, A–C, and Supplemental Table 1; supplemental material available online with this article; https://doi.org/10.1172/jci.insight.190445DS1) (17, 19, 20). PLWH were without neuropsychiatric confounders and underwent comprehensive neurocognitive testing (21).

The neuropsychological evaluation assessed 7 cognitive domains associated with HIV-associated neurocognitive disorder (HAND) (attention/working memory; processing speed; learning; recall; abstraction/executive functioning; verbal fluency; and motor skills) adapted from Rippeth et al. (22) and previously used by our group (21). Raw scores were age-, education-, sex-, and ethnicity-corrected where appropriate; transformed into T-scores; and then converted to deficit scores. The Global Deficit Score (GDS) was calculated by summing deficit scores and dividing by the number of administered tests, providing a continuous measure of cognition with established sensitivity and specificity for HAND classification.

Participant age significantly correlated with the estimated duration of HIV-1 infection in our cohort. The GDS did not correlate with age or duration of HIV-1 infection, reflecting the fact that multiple, complex factors drive NCI in PLWH (Supplemental Figure 1, A–C).

To generate dermal fibroblast cultures from well-characterized and neurocognitively tested PLWH, skin samples from all 6 PLWH and 2 of 8 PWOH were collected via skin punch biopsy (Supplemental Table 1). Dermal fibroblasts were isolated from skin biopsy samples as detailed in Methods. Dermal fibroblast cultures from the remaining 6 PWOH were obtained from a public repository.

To generate iNs, all participant-derived dermal fibroblasts were transduced with a lentiviral vector (UNA vector) (19) for doxycycline-dependent expression of neuronal transcription factors NGN2 (NEUROG2) and ASCL1. Transduced fibroblasts were termed UNA fibroblasts prior to initiating transdifferentiation to account for lentiviral transduction-mediated genetic modification (Supplemental Figure 1D). Treatment of UNA fibroblasts with doxycycline and a cocktail of differentiation factors for 21 days resulted in a mixed population of neurons and non-converted cells as observed by light microscopy and as previously described (Supplemental Figure 1E) (20).

To isolate bona fide iNs from this mixed population, we performed fluorescence-activated cell sorting (FACS) targeting polysialylated-neural cell adhesion molecule (PSA-NCAM) on live (DAPI^–^) cells (Supplemental Figure 1D). To assess purity and obtain first insights into neuronal gene expression, single-cell RNA (scRNA) analysis was conducted with iNs from 2 PWOH (Figure 1, D–G). To maximize cohort representation, we selected iNs from a young male donor (27 years) and an older female donor (66 years). This scRNA analysis revealed a small subset of cells with a putative fibroblast-associated transcriptome, indicated by expression of collagens *COL1A1*, *COL3A1*, and *COL4A1* (Figure 1, E and G). Nevertheless, the majority of cells expressed neuronal marker genes *TUJ1* (also *TUBB3*), *MAP2*, and *MAPT* (tau) while lacking fibroblast marker expression (Figure 1, F and G). Thus, using these 2 distinct donors for scRNA analysis, we demonstrated that this protocol yields high rates of neurons irrespective of donor age or sex.

Importantly, immunocytochemistry at 3 days post-FACS confirmed TUJ1 and MAP2 protein expression and clearly showed neuronal morphology as depicted for cells derived from donor 100-O3, representative of the entire cohort (Figure 1, H and I, and Supplemental Figure 1F). This result was in concordance with a prior study from an independent research group following the same protocol, which obtained approximately 90% TUJ1-positive cells by immunocytochemistry (14).

In addition to revealing pan-neuronal marker gene expression in our iNs, scRNA analysis confirmed previous findings of 2 subpopulations within the iNs: a larger subset of potentially glutamatergic (*SLC17A7*^+^) and smaller subset of potentially GABAergic (*GAD1*^+^) neurons, with virtually no overlap of expression (Supplemental Figure 2, A and B). Our analysis further confirmed lack of choline O-acetyltransferase (*CHAT*) and tryptophan hydroxylase 2 (*TPH2*) expression, indicating absence of cholinergic or serotonergic neurons, respectively (Supplemental Figure 2A).

To validate the transdifferentiation protocol across the entire cohort, we conducted differential gene expression analysis with bulk RNA isolated from iN samples and their matched UNA fibroblasts derived from all donors (PLWH and PWOH). We harvested RNA while matching samples (iNs and UNA fibroblasts) were cultured at the same passage to limit long-term cell culture–mediated effects. As a result, the only difference between UNA fibroblasts and their matched iNs was 21 days of transdifferentiation and subsequent cell sorting. The conversion protocol did not yield sufficient iNs from PWOH donor 3007 for bulk-RNA analysis. Therefore, only UNA fibroblasts from donor 3007 were included in the following analysis.

**Figure 2:**
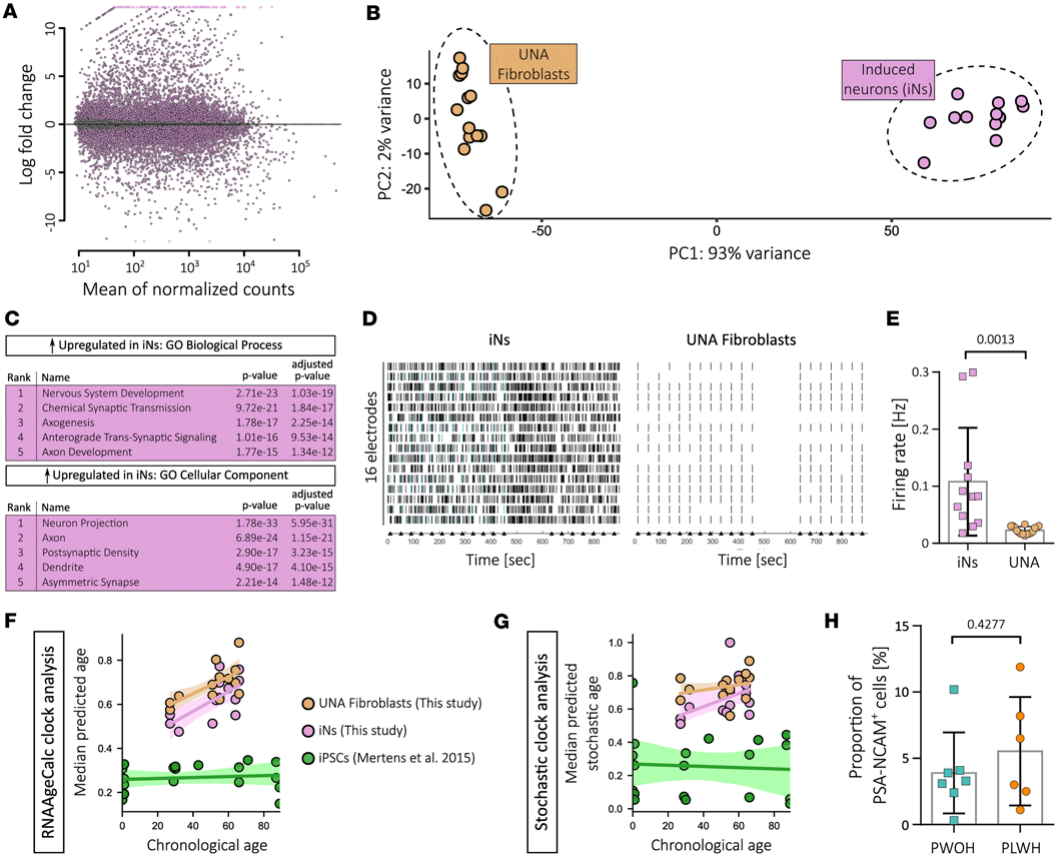
iNs show neuronal gene expression and action potential firing and retain the biological age of their donors. (**A**) MA plot based on bulk RNA sequencing showing differential gene expression of all iN samples after sorting for PSA-NCAM^+^ cells compared with UNA fibroblasts. (**B**) PCA plot showing clustering of iN- and UNA fibroblast–derived RNA samples after bulk analysis. (**C**) Top 5 ranked gene ontology (GO) terms of biological processes and cellular components associated with the significantly upregulated genes in iNs compared with UNA fibroblasts. (**D**) Raster plots showing electrical activity of iNs (left) or UNA fibroblasts (right) from donor 100-O1 analyzed by multi-electrode array. Vertical bars in black represent electrode spikes and in blue single-channel bursts (at least 5 spikes/s). Synchronized electrical-pulse (arrowheads; 500 mV for 100 μs, every 40 seconds) stimuli were applied via all electrodes to test for evoked activity. (**E**) Mean firing rate of iNs and UNA fibroblasts (including artifacts due to the applied electrical pulses). Statistical significance tested with unpaired, 2-tailed *t* test. Data presented as individual data points with mean ± SD and *P* value. (**F** and **G**) Median predicted transcriptomic age (scaled 0–1) across 16 RNAAgeCalc clocks (**F**) and of an ensemble of 100 stochastic clocks (**G**) plotted against chronological age for human iPSCs (green), UNA fibroblasts (brown), and iNs (pink). Transcriptomic data for the analysis of UNA fibroblasts and iNs were generated during this study, and data of iPSCs as control for age reset were derived from Mertens et al. 2015 (EMBL-EBI ArrayExpress: E-MTAB-3037) using publicly available data on healthy donors (*n* = 19) (17). (**H**) Proportion of PSA-NCAM^+^ cells between the 2 groups determined by flow-activated cell sorting analysis at day 21 of neuronal conversion. Statistical significance tested with unpaired, 2-tailed *t* test. Data presented as individual data points with mean ± SD and *P* value.

Differential gene expression analysis showed that the morphological transition from fibroblasts to neuronal phenotype was accompanied by drastic transcriptional changes with over 10,000 genes differentially expressed in iNs compared with UNA fibroblasts, illustrated in a log ratio versus average expression (MA) plot (*P*-adj. < 0.05, log2fc > ± 0.5) (Figure 2A and Supplemental Figure 3). We performed principal component analysis (PCA) to confirm that the transcriptomic signature of all iN samples is comparable to the 2 samples whose neuronal identity was previously confirmed via scRNA analysis (samples 3962 and 4642). PCA reduces high-dimensionality sequencing data to 2 dimensions, plotted on *x* and *y* axes, while preserving as much of the original variation as possible. iN and UNA fibroblast samples formed 2 distant clusters, with all iN samples clustering with samples 3962 and 4642 (Figure 2B and Supplemental Figure 2C, blue labels). Overall, this PCA based on all iN samples following the scRNA analysis with iNs from donors 3962 and 4642 strongly indicated that all iN samples exhibit the same degree of neuronal identity and cellular composition.

Two fibroblast cultures from PWOH participants 100-O1 and 100-O3 were generated in our laboratories with the PLWH-derived fibroblast cultures following in-house protocols (Supplemental Table 1). Fibroblast cultures from the remaining 6 PWOH were obtained from a public repository. Therefore, we analyzed whether this circumstance affected their gene expression profile, which could affect downstream iN gene expression analysis. We investigated how UNA fibroblast samples 100-O1 and 100-O3 clustered in the PCA plot. UNA fibroblast samples from participants 100-O1 and 100-O3 clustered with remaining PWOH samples from the public repository (Supplemental Figure 2C), indicating that fibroblast source did not affect gene expression profile sufficiently to confound downstream differential gene expression analysis between PLWH- and PWOH-derived cells.

To verify the neuronal state of iNs, we performed gene set enrichment analysis (GSEA) with the significantly differentially expressed genes (DEGs) to determine gene ontology (GO) terms associated with UNA fibroblast-to-iN transdifferentiation. As expected, GO terms of biological processes associated with upregulated genes after transdifferentiation were linked to neuronal development and function (Figure 2C). This was corroborated by top GO terms for cellular components, corresponding to neuron-specific compartments (Figure 2C). Downregulated genes following transdifferentiation into iNs were associated with GO terms corresponding to fibroblast function and related cellular compartments, underlining loss of fibroblast-associated characteristics over 21 days of transdifferentiation (Supplemental Figure 2D). Together, this strongly supported transdifferentiation of participant-derived fibroblasts into neurons.

To test whether morphological and transcriptomic validation of the neuronal state is accompanied by neuronal activity (i.e., action potential firing), we analyzed whether transdifferentiation of donor-derived skin fibroblasts leads to electrical activity of produced iNs. For this, UNA fibroblasts and iNs from the same donor were cultured on Axion microelectrode array (MEA) plates for extracellular field recordings, and electrical firing behavior was analyzed while stimulating with electrical pulses (500 mV for 100 μs every 40 seconds), with a pause in stimulation to test for both evoked and spontaneous action potential firing. iNs showed spontaneous and evoked action potential firing whereas electrodes covered by UNA fibroblasts detected no electrical signals beyond stimulation artifacts (Figure 2, D and E). We therefore confirmed that generated iNs fire action potentials.

We also confirmed that transdifferentiation of primary fibroblasts into iNs retained the biological age of donor-derived cells as previously demonstrated (14, 17, 23). We applied 16 distinct transcriptomic aging clocks from the RNAAgeCalc package (see Methods) (24), each predicting biological age from transcriptomic data using variations in feature selection and reference datasets. Using this approach, we analyzed publicly available transcriptomic data of donor-derived fibroblasts and iNs from Mertens et al. 2015 (17) and our data on donor-derived UNA fibroblasts and iNs, using donor-derived iPSC data from the literature (17) as control for the effect of erasing aging signatures. Across all clocks, iNs displayed elevated predicted transcriptomic age compared with iPSCs and showed age-related trajectories resembling those of fibroblasts, which was particularly clear in our dataset (Figure 2F and Supplemental Figure 4A). Mixed model estimates confirmed significant age effects in both iN datasets (our data FDR = 1.24 × 10^–4^; Mertens data FDR = 2.86 × 10^–3^), but not in iPSCs (FDR = 0.985), supporting the observation that iPSC reprogramming erases donor-derived aging signatures, while direct conversion to iNs preserves them (Supplemental Figure 4B). The consistent pattern in both newly generated and public datasets underscores robustness and reproducibility across cohorts.

To independently validate transcriptomic age predictions beyond signature-based clocks, we employed stochastic clocks that estimate biological age by quantifying accumulation of random transcriptomic variation over time (see Methods) (25). Unlike aging clocks trained on biological data, the stochastic clock captures age-related patterns through simulated noise accumulation, operating on a different conceptual basis. Applying an ensemble of 100 independently trained stochastic clocks, we observed that iNs consistently exhibited a significantly higher predicted stochastic age than iPSCs, with trajectories paralleling those of donor-derived fibroblasts (Figure 2G and Supplemental Figure 4, C and D). These results recapitulated RNAAgeCalc-based results and support reproducibility of biological age retention in iNs across distinct predictive models. Together, both biological and stochastic aging clocks support the conclusion that directly induced neurons preserve transcriptomic hallmarks of aging from donor-derived fibroblasts, whereas iPSC reprogramming erases such signatures. This reinforces the utility of iNs as a model system for studying cellular aging in donor-specific contexts and supports their suitability to identify potential differences in iN gene expression between PLWH and PWOH.

Finally, we investigated whether transdifferentiation efficiency between PLWH- and PWOH-derived cells differed, which could potentially confound subsequent differential gene expression analysis. Therefore, we compared rates of PSA-NCAM–positive cells at flow-activated cell sorting 21 days after starting neuronal conversion. There was no significant difference in transdifferentiation efficiency between PLWH-derived and PWOH-derived cells (Figure 2H). Hence, we did not expect confounding effects of transdifferentiation efficiency on differential gene expression analysis.

In summary, these analyses demonstrate successful execution of the previously published protocol for generating participant-derived iNs from our cohort of 6 PLWH and 7 matched PWOH controls. Hence, we obtained neurons via a protocol that preserves age-related characteristics and was previously demonstrated to also preserve disease state–associated biological changes. This allows us to reveal biologically plausible neuronal differences between virologically suppressed PLWH and PWOH.

### PLWH-derived iNs exhibit statistically significant DEGs compared with iNs from PWOH.

**Figure 3:**
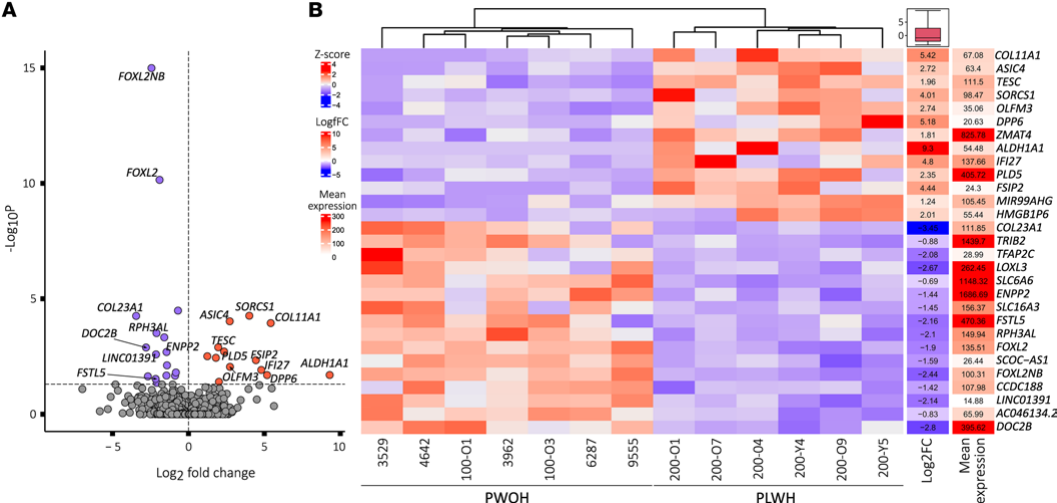
PLWH-derived iNs exhibit statistically significant DEGs compared with iNs from PWOH. (**A**) Volcano plot showing the 29 statistically significant (*P*-adj. < 0.05, log2fc > ± 0.5) DEGs in PLWH-derived iNs compared with PWOH-derived iNs following bulk RNA analysis with iNs. *Y* axis plots the *P*-adj. values, and the dotted line indicates the selected cutoff of *P*-adj. < 0.05. (**B**) Heatmap showing the clustering of PLWH- vs. PWOH-derived iN RNA samples based on expression levels of the 29 DEGs while displaying the log_2_ fold change and mean expression.

We aimed to investigate if chronic HIV-1 infection in the context of concurrent treatment with cART affects the gene expression signature of the iNs. We used bulk RNA-sequencing data to compare gene expression profiles of iNs derived from PLWH to those from PWOH. This transcriptome analysis identified 29 DEGs between PLWH- and PWOH-derived iNs (*P*-adj. < 0.05, log2fc > ± 0.5) (Figure 3, A and B, and Supplemental Table 2). Of these, 13 genes were upregulated, and 16 genes were downregulated in iNs derived from PLWH.

**Figure 4:**
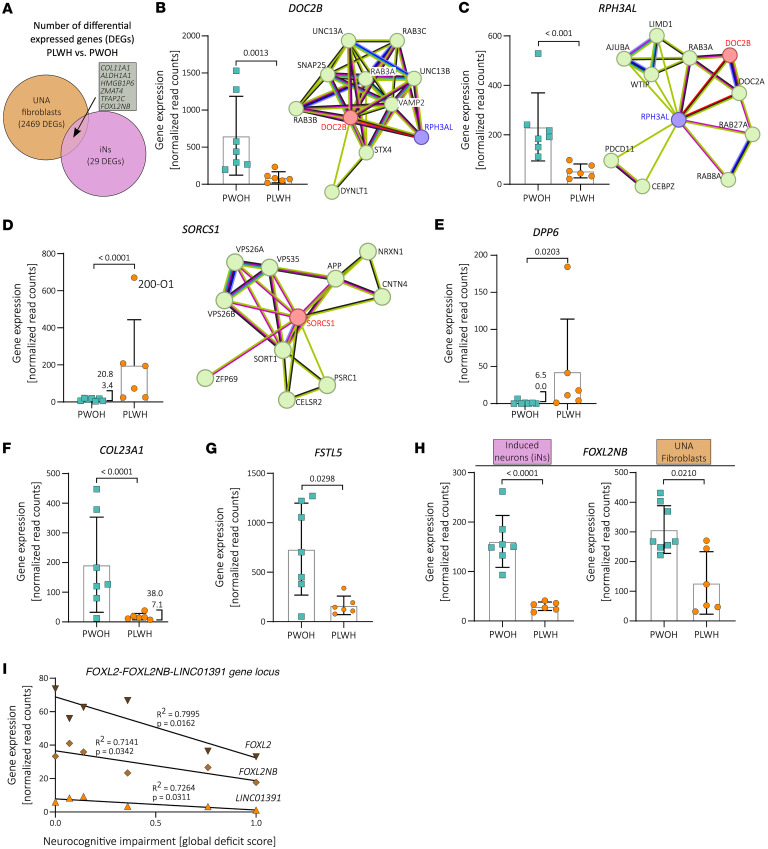
Differential gene expression of candidate genes potentially affected in PLWH-derived iNs. (**A**) Venn diagram showing the numbers of DEGs between the PLWH- and PWOH-derived UNA fibroblasts and iNs. The gene symbols of the 6 genes that are differentially expressed in both cell types are displayed in the gray box. (**B**–**E**) Gene expression levels in PLWH- vs. PWOH-derived iNs and STRING protein association networks of *DOC2B* (**B**), *RPH3AL* (**C**), and *SORCS1* (**D**) (33, 34). (**E**–**G**) Gene expression levels in PLWH- vs. PWOH-derived iNs of *DPP6* (**E**), *COL23A1* (**F**), and *FSTL5* (**G**). (**H**) Gene expression levels in PLWH- vs. PWOH-derived iNs and UNA fibroblasts of *FOXL2NB*. (**B**–**H**) Data presented as individual data points with mean ± SD and *P*-adj. value derived from the conducted Wald test corrected for false discovery rates (FDR) using the Benjamini-Hochberg method (see Methods section). (**I**) Correlation of gene expression levels of *FOXL2*, *FOXL2NB*, and *LINC01391* with neurocognitive impairment in the PLWH study group (*n* = 6). Data points are individual values and lines depict linear regression functions.

To assess whether this finding of 29 DEGs between iNs from PLWH and PWOH may have been affected by an interaction between chronic HIV-1 infection or cART on fibroblast biology, and thus cellular reprogramming, we also analyzed differential gene expression between UNA fibroblasts derived from PLWH and PWOH. We found 2,469 DEGs between UNA fibroblasts (*P*-adj. < 0.05, log2fc > ± 0.5) (Figure 4A and Supplemental Table 3). Downregulated genes were associated with mitotic processes while upregulated genes were not significantly associated with any biological process (Supplemental Table 4). We were unable to compare identified DEGs with previously reported datasets since comparable datasets of skin fibroblast transcriptomes from virally suppressed PLWH compared with age- and sex-matched controls are lacking.

The high number of DEGs between the UNA fibroblast samples did not affect transdifferentiation efficiency in our cohort as previously shown (Figure 2H). Moreover, we identified 23 genes differentially expressed only in iN samples (Figure 4A). As a result, we considered these 23 DEGs to be iN specific in our setting (i.e., after comparing donor-derived UNA fibroblasts and iNs). This list of 23 DEGs contained several genes including *DOC2B*, *RPH3AL*, *SORCS1*, and *DPP6* that are associated with known or presumed neuronal functions, as well as *IFI27*, which has been previously found to be differentially expressed in autopsy tissue samples from the NeuroAIDS Tissue Consortium comparing PLWH and PWOH (see below) (26–30). These candidates may present pathways relating to neuronal function in virologically suppressed PLWH.

### Differential expression of genes related to synaptic transmission pathways in PLWH iNs compared with PWOH iNs.

Expression of the double C2 domain beta (*DOC2B*) gene was reduced 6.95-fold in PLWH compared with PWOH on average (Figure 4B). *DOC2B* is readily expressed in human brain and important for neuronal activity (31). DOC2B has been identified as a cytosolic Ca^2+^ sensor mediating spontaneous neurotransmitter release (26). Double knockdown of the double C2 domain proteins DOC2A and DOC2B results in decreased spontaneous transmitter release from hippocampal neurons, which could be rescued by expressing DOC2B (26). A subsequent study described its role in hippocampal synaptic plasticity (32). DOC2B interacts with several proteins important for neurotransmitter release, including SNARE complex components as shown by pulldown analysis (26) and STRING network analysis (Figure 4B) (33, 34). Interestingly, the network analysis also depicted a link to rabphilin 3A like (without C2 domains) (*RPH3AL*, previously *Noc2*), another gene significantly downregulated in PLWH- compared with PWOH-derived iNs (Figure 4C). This co-reduction may be due to shared gene regulatory elements supposedly controlling *DOC2B* and *RPH3AL* transcription on chromosome 17 (35, 36). RPH3AL is a Rab effector protein associated with secretory vesicle release (27), expressed presumably at low levels in the brain (28). A recent genome-wide association study (GWAS) showed that the *RPH3AL* missense mutation rs117190076 increases risk for late-onset AD (37). Hence, downregulation of the genomic locus containing *DOC2B* and *RPH3AL* may affect synaptic vesicle release in 2 different ways.

In contrast with *DOC2B* and *RPH3AL*, olfactomedin 3 (*OLFM3*) showed increased expression in iNs from PLWH compared with PWOH (Figure 3, A and B). OLFM3 protein is expressed in cortical and hippocampal neurons (38). It has been shown to bind different subunits of the postsynaptic AMPA receptor (α-amino-3-hydroxy-5-methyl-4-isoxazole-propionic acid receptor), namely GRIA1 and GRIA2, and its overexpression in mouse hippocampus affects their membrane expression (38). In this context, increased OLFM3 expression has been linked to epilepsy because it was suggested to alter AMPA receptor activity (38).

### Expression of AD-associated SORCS1 is increased in PLWH-derived iNs.

We observed significantly elevated *SORCS1* expression levels in iNs from PLWH when compared with PWOH iNs (Figure 4D), indicating a role for *SORCS1* gene expression in HIV-related NCI. In addition to apolipoprotein E, amyloid-beta, and tau protein (39, 40), sortilin related VPS10 domain containing receptor 1 (*SORCS1*) expression is among the best-known risk factors in AD. SORCS1 is associated with different components of the amyloid-beta pathway and has been specifically shown to play a role in aberrant amyloid precursor protein (APP) trafficking (Figure 4D) (29, 41). SORCS1 is a general regulator for intracellular trafficking and is important for maintaining neuronal functionality by, for instance, sorting the AMPA glutamate receptor and synaptic adhesion molecule neurexin (NRXN), which ensures proper glutamatergic transmission (42).

Interestingly, the association between *SORCS1* single nucleotide polymorphisms (SNPs) and AD appears to exhibit sexual dimorphism, with stronger correlation observed in women (40, 43). In our cohort of PLWH, *SORCS1* expression was 3-fold higher in iNs from the participant of female sex (200-O1) compared with iNs from the participant with the second highest *SORCS1* expression (Figure 4D). However, the limited sample size prevents us from drawing conclusions regarding the effect of sex on *SORCS1* expression.

### Expression of the potassium ion channel auxiliary factor DPP6 is increased in iNs derived from PLWH.

Dipeptidyl peptidase like 6 (*DPP6*) exhibited increased expression levels in iNs from PLWH compared with PWOH (Figure 4E). DPP6 RNA and protein expression throughout the human body is predominantly found within the brain with low region specificity (available from v23.0 Protein Atlas; https://www.proteinatlas.org/ENSG00000130226-DPP6) (44, 45). It is an important auxiliary factor of potassium ion (K^+^) channels, and its expression is associated with synaptic function and impairments in learning and memory (30, 46–48). The NHGRI-EBI GWAS catalog (49) includes *DPP6* SNPs associated with cognitive decline (GCST009443; GCST90308745) (50, 51) and hippocampal volume (GCST90104700) (52). Despite obtaining low average expression of *DPP6* in our assay, the bulk RNA sequencing resulted in 0 reads for *DPP6* RNA in 4 out of 7 iN samples from PWOH whereas *DPP6* expression was detected in all PLWH iN samples (Figure 4E). A schizophrenia study that generated participant-derived iPSC neurons likewise found increased *DPP6* transcript levels in neurons from schizophrenia compared with healthy controls, an observation mechanistically linked to decreased neuronal activity (53).

### Differential expression of ECM-associated genes in iNs from PLWH compared with those from PWOH.

Extracellular matrix (ECM) proteins are important for neuronal development and function (54), and their dysregulation has been linked to neurodegeneration (55). In this study, iNs from PLWH exhibited significant differential expression of several ECM-associated proteins compared with iNs derived from PWOH. In most cases, similar changes in gene expression patterns were not noted in matched UNA fibroblast samples, indicating neuron-specific effects.

Collagen type XXIII alpha 1 chain (*COL23A1*) is an ECM-associated protein that may reveal mechanic insights into HIV-related NCI. Its expression was more than 8-fold reduced in iNs from PLWH compared with PWOH (Figure 4F). Further, we obtained raw read counts for *COL23A1* RNA via bulk RNA sequencing from only 4 UNA fibroblast samples ranging from 1 to 12 raw reads, substantiating iN-specific *COL23A1* expression in our assay (data not shown). Indeed, *COL23A1* expression is found across human brain in multiple cell types, including neurons and astrocytes (available from v23.0 Protein Atlas; https://www.proteinatlas.org/ENSG00000050767-COL23A1/brain) (44, 45). COL23A1 is a type II membrane protein belonging to the transmembrane collagen family (56), and despite limited knowledge of its role in neural function, SNPs of its gene are associated with the rate of cognitive decline in AD (GCST010567) (57) and memory performance (GCST90104696) (52).

In addition to *COL23A1*, PLWH-derived iNs exhibited differential expression of the collagen family member *COL11A1* (collagen type XI alpha 1 chain) (Figure 3B). While potentially of interest, unlike *COL23A1*, increased *COL11A1* expression in PLWH samples compared with PWOH controls was not restricted to iNs and was also observed in UNA fibroblasts (Figure 4A). Of note, *COL11A1* is expressed in brain and skin (available from v23.0 Protein Atlas; https://www.proteinatlas.org/ENSG00000060718-COL11A1) (44, 45).

Besides structural proteins like collagen family members, different secreted proteins are also associated with the ECM. Our iNs from PLWH showed decreased *ENPP2* expression (Figure 3B). The *ENPP2* gene encodes ectonucleotide pyrophosphatase/phosphodiesterase 2, better known as autotaxin. Autotaxin is an enzyme secreted into the ECM by different cell types throughout the human body, including neural cells (58). Autotaxin exerts biological functions by processing lysophosphatidylcholine into lysophosphatidic acid (LPA), which then binds to one of its several G protein–coupled receptors (LPA1–LPA6) (58). Interestingly, LPA signaling is involved in numerous physiological processes, including neurogenesis, neuronal differentiation, synapse formation, migration, and cortical development (58). Hence, decreased *ENPP2* expression in neural cells may affect brain functioning via dysregulated LPA signaling.

Follistatin like 5 is also a secreted protein expressed throughout human brain, predominantly in cerebellum, in inhibitory and excitatory neurons (available from v23.0 Protein Atlas; https://www.proteinatlas.org/ENSG00000168843-FSTL5) (44, 45). Although its role in physiological brain processes is not well described, SNPs within the *FSTL5* gene are associated with general cognitive ability (GCST006269) (59), dementia and AD in non-APOE ε4 allele carriers (GCST90244035; GCST90244033) (60), and working memory (GCST006930) (61) as annotated in the NHGRI-EBI GWAS catalog (downloaded October 3, 2024) (49). We found that *FSTL5* expression was on average 4.4-fold decreased in PLWH-derived iNs compared with PWOH iNs (Figure 4G).

### Protein-protein interaction network mapping supports differential ECM organization and synaptic transmission in PLWH iNs and indicates neuronal apoptosis as another affected pathway.

We investigated whether protein-protein interaction (PPI) network mapping reveals insights into affected cellular pathways in PLWH-derived iNs. We determined first-order interaction partners for gene products of the 29 DEGs between PLWH- and PWOH-derived iNs using the IntAct Molecular Interaction Database (62) and The Human Reference Interactome (HuRI) (Supplemental Figure 5A and Supplemental Table 5).

GSEA revealed that biological processes associated with the resulting PPI network were related to neuronal apoptosis (Supplemental Figure 5B and Supplemental Table 6). Further, this analysis suggests that ECM organization is affected in iNs derived from PLWH compared with PWOH (Supplemental Figure 5B). In addition to the ECM, PPI network analysis substantiated an influence of the 29 DEGs on synaptic transmission (Supplemental Figure 5B).

When analyzing the obtained PPI network with regard to associated diseases, it was therefore not surprising to find associations with terms like neurodegenerative disease, dementia, or AD (Supplemental Figure 5B) (63, 64).

### Expression of the FOXL2NB-FOXL2-LINC01391 genome locus is reduced in PLWH-derived iNs and associated with the degree of NCI.

Expression of the FOXL2 neighbor gene (*FOXL2NB*, previously *C3orf72*) was significantly downregulated in PLWH- compared with PWOH-derived iNs and also in UNA fibroblasts. However, this effect was more pronounced in iN samples (Figure 4H). This suggests that differential *FOXL2NB* expression between PLWH and PWOH in iN samples is not an experimental artifact mediated by choice of our original cell type (participant-derived primary dermal fibroblasts), but rather a cell type–independent effect that appears more prominent in neurons than in fibroblasts.

Furthermore, expression levels of the transcription factor forkhead box L2 gene (*FOXL2*), and the long non-coding RNA *LINC01391*, were significantly reduced only in PLWH iN samples and not in PLWH UNA fibroblasts. We found this of particular interest because the 3 genes (*FOXL2NB*, *FOXL2*, and *LINC01391*) are located in close proximity to each other on human chromosome 3, and their expression is controlled by shared gene regulatory elements as annotated in the *GeneHancer* database (Supplemental Figure 6) (35, 36). Thus, transcription rate at this genomic locus may be decreased in neurons of PLWH. Interestingly, little is known about the function of *LINC01391* and *FOXL2NB* in brain, but *FOXL2* has been recently associated with AD (65, 66).

Moreover, expression levels of *FOXL2NB*, *FOXL2*, and *LINC01391* showed a negative correlation with the GDS in our cohort (Figure 4I). This means that higher NCI scores were associated with lower expression of the *FOXL2NB*-*FOXL2*-*LINC01391* genome locus in iNs from PLWH. Only 2 PWOH underwent neurocognitive testing in our study, precluding assessment of this correlation in controls.

### Autopsy tissue samples from the NeuroAIDS Tissue Consortium verify increased levels of interferon alpha-inducible protein 27 in the brains of PLWH.

Sustained inflammation of the CNS is considered a major factor in the development of HIV-related NCI. Several genes of the inflammatory signaling cascade have been identified that may contribute to this, including *ISG15*, *IFIT1*, *IFI44*, and *IFITM1* (8, 67–69).

**Figure 5:**
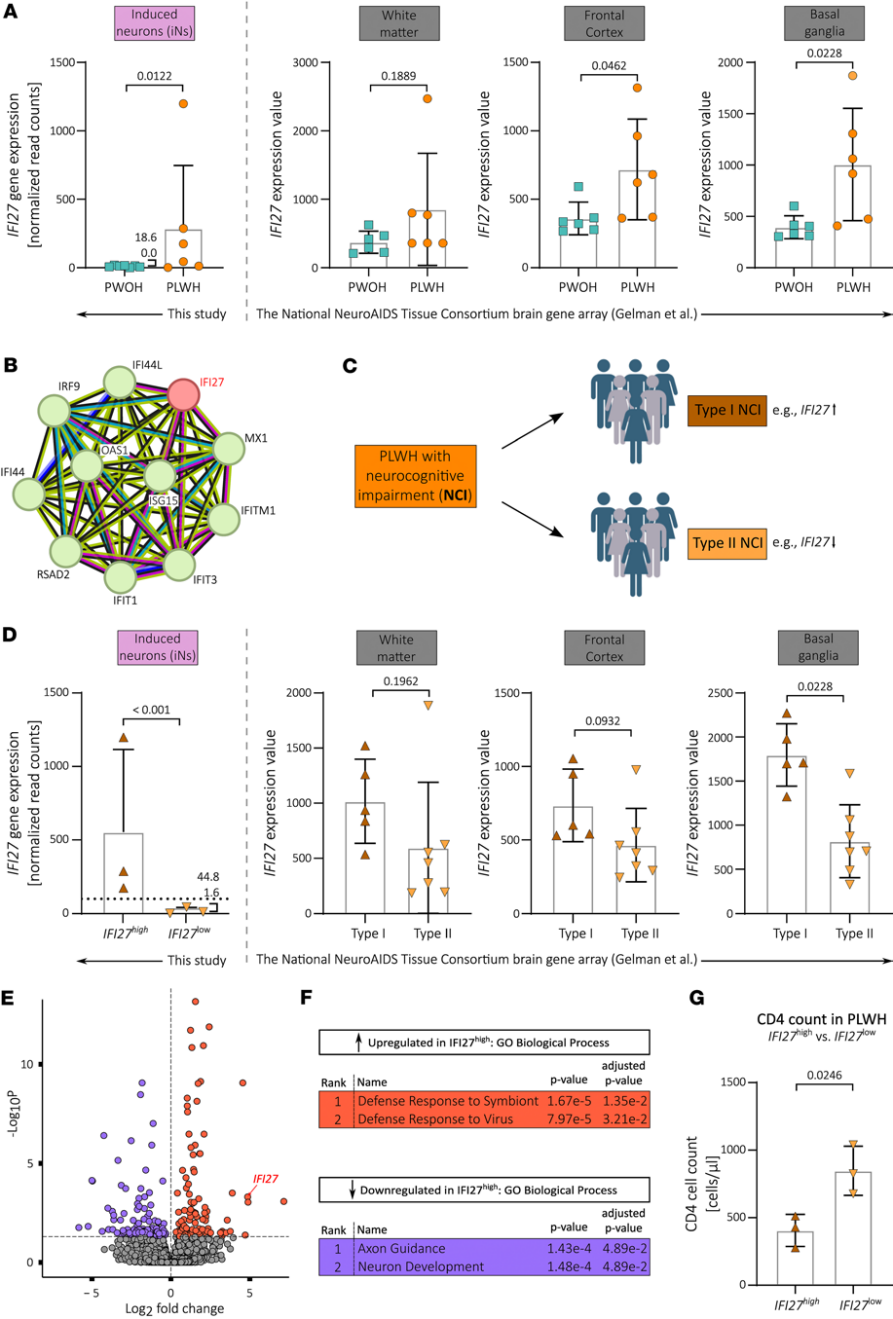
*IFI27* expression levels are increased in PLWH-derived iNs and postmortem brain tissue samples compared with PWOH-derived samples. (**A**) *IFI27* gene expression levels in PLWH- vs. PWOH-derived iNs and postmortem brain tissue samples. (**B**) STRING protein association network of *IFI27* (33, 34). (**C**) Scheme illustrating the concept of type I and II neurocognitive impairment in PLWH according to Gelman et al. together with the associated *IFI27* expression (8). (**D**) *IFI27* gene expression levels in PLWH *IFI27*^hi^ vs. *IFI27*^lo^ iNs and type I vs. type II PLWH-derived postmortem brain tissue samples. (**E**) Volcano plot showing the statistically significant (*P*-adj. < 0.05, log2fc > ± 0.5) DEGs in PLWH *IFI27*^hi^ vs. *IFI27*^lo^ iNs based on our bulk RNA analysis. (**F**) Top 2 ranked GO terms of biological processes associated with the significantly up- and downregulated genes in PLWH *IFI27*^hi^ vs. *IFI27*^lo^ iNs. (**G**) CD4^+^ T cell counts in PLWH divided into *IFI27*^hi^ vs. *IFI27*^lo^ participants. Statistical significance tested with unpaired, 2-tailed *t* test (**A**, **D**, and **G**) or derived from the conducted Wald test corrected for FDR using the Benjamini-Hochberg method on whole-transcriptome data (**A** and **D**). Data presented as individual data points with mean ± SD. *IFI27* expression values in postmortem brain tissue samples derived from Gelman et al. (8).

Interestingly, our differential gene expression analysis revealed interferon alpha-inducible protein 27 (*IFI27*) to be significantly upregulated in iNs from virologically suppressed PLWH compared with PWOH (Figure 5A). This *IFI27* upregulation was not observed in UNA fibroblasts and was therefore considered iN specific in our study design. STRING network analysis clearly showed that *IFI27* was closely associated with *ISG15*, *IFIT1*, *IFI44*, *IFITM1*, and many additional genes of the inflammatory pathway (Figure 5B).

To analyze whether PLWH-derived iNs exhibit a gene expression profile indicative of a broader inflammatory response that may have been masked by our small cohort size, we performed unfiltered GSEA using GSEA 4.3.3 (70). Applying GSEA to the differential gene expression profile of PLWH-derived iNs showed a significant association with the hallmark gene set interferon-α response (FDR *q* value 0.11; *P* value 0.026, NES 1.462) (Supplemental Figure 7A) (71, 72). This was based on upregulation of inflammatory genes, such as *ISG15*, *IFI44L*, *IFI44*, and *IFITM1*, which contributed to positive core enrichment during GSEA (Supplemental Figure 7B).

*IFI27* was the only significantly upregulated inflammatory response-related gene in our cohort. Given its strong connection to inflammatory genes previously linked to HIV-related NCI (Figure 5B), we mined the literature to find preexisting evidence of *IFI27* involvement in HIV-related brain pathology. Importantly, we found 3 independent studies comparing gene expression in postmortem brain samples between PLWH and PWOH that found significantly increased *IFI27* expression in autopsy brain tissue from PLWH (8, 68, 73). Study characteristics and methodologies are provided (Supplemental Table 7). While these studies analyzed total gene expression from gross brain tissue (i.e., not only neuronal gene expression), these findings support the relevance of the iN model to study HIV-related NCI.

Solomon et al. compared gene expression in frontal white matter tissue between 34 PLWH (≥45 years old) on cART and 24 age-matched PWOH via gene expression profiling (NanoString nCounter technology) with 933 selected probes and showed a more than 2-fold increase in *IFI27* expression in the PLWH-derived brain tissue samples (Supplemental Table 7) (68).

Gabuzda et al. recently performed gene expression profiling (NanoString nCounter technology) using a selected set of 78 probes representing previously described HIV-1–associated genes (e.g., based on Solomon et al.) (68) and found 1.73-fold increased *IFI27* expression in frontal lobe white matter samples from 28 PLWH (≥40 years old) on cART compared with samples from 20 age- and sex-matched PWOH (Supplemental Table 7) (73).

Last, Gelman et al. performed a gene expression array with postmortem tissue from frontal cortex (neocortex), white matter, and basal ganglia (neostriatum) obtained from the National NeuroAIDS Tissue Consortium in 2012 (8). By analyzing their publicly available gene array dataset on postmortem brain tissue, we observed that *IFI27* expression levels were significantly elevated in the basal ganglia and frontal cortex of PLWH without NCI when compared with PWOH (Figure 5A and Supplemental Table 7). The increase in *IFI27* expression observed for analyzed white matter samples was not significant but nevertheless showed a trend toward upregulation (Figure 5A). Together, this shows that *IFI27* expression is increased in the brains of PLWH.

Based on a comparison of DEGs between PWOH and PLWH with NCI that either developed HIV encephalitis (HIVE) or did not, Gelman and colleagues suggested the presence of 2 distinct pathomechanisms that lead to NCI in PLWH irrespective of cART: with inflammatory changes (type I NCI) and without inflammatory changes (type II NCI) (Figure 5C). Type I and II NCI in PLWH supposedly underlie different biological pathways, and several marker genes, including *IFI27*, were identified whose expression levels together could distinguish between the 2 types (8, 74).

In this regard, *IFI27* expression alone may not be enough to reliably distinguish between the 2 types because its expression differed significantly only in basal ganglia samples (Figure 5D). However, to test whether the distinction of type I and II NCI may be preserved after transdifferentiation of dermal fibroblasts into iNs, we divided our PLWH study group into *IFI27*^lo^ versus *IFI27*^hi^ participants (Figure 5D). For this classification, we applied the average fold-change between *IFI27* expression in PWOH versus PLWH with type I NCI in postmortem basal ganglia samples (Figure 5A, basal ganglia, left bar; versus Figure 5D, basal ganglia, left bar) as a cutoff, which is approximately 5-fold. Hence, we chose a cutoff for grouping PLWH-derived iNs that was 5-fold the average *IFI27* expression in iNs derived from PWOH (=51.50 normalized read counts) (Figure 5D). iN samples with *IFI27* expression above this cutoff were considered *IFI27*^hi^, while those below were considered *IFI27*^lo^.

Next, we performed differential gene expression analysis to compare the transcriptional profile of *IFI27*^hi^ with *IFI27*^lo^ PLWH iNs, the first now serving as a putative surrogate model for type I NCI (*IFI27*^hi^) and the latter for type II NCI neurons (*IFI27*^lo^). We identified 215 DEGs (*P*-adj. < 0.05, log2fc > ± 0.5), of which 106 were downregulated and 109 upregulated in *IFI27*^hi^ PLWH iNs (Figure 5E, Supplemental Figure 8, and Supplemental Table 8). The resulting PCA plot did not show distinct clustering of the 2 groups, indicating that while the groups show differential gene expression profiles, the effect size may not be large (Supplemental Figure 9A). This observation might have been due to the low sample size.

GSEA showed that a significant number of upregulated genes were associated with antiviral defense (Figure 5F). This finding is consistent with expected upregulation of inflammatory response genes when grouping based on *IFI27* expression. These results align with Ingenuity Pathway Analysis performed by Gelman and colleagues, who found canonical pathways of antiviral defense mechanisms upregulated in their type I NCI samples (8). Importantly, GO terms associated with downregulated genes in *IFI27*^hi^ PLWH iNs were related to neuronal development and formation of neuronal processes, with the top-ranked GO term being axon guidance, a term likewise found to be associated with downregulated genes in type I NCI samples in the study by Gelman et al. (Figure 5F) (8). Hence, our analysis indicated that studying biological pathways underlying the distinction of type I and II NCI may be possible by transdifferentiation of participant-derived dermal fibroblasts into iNs.

Last, to investigate whether the distinction between *IFI27*^hi^ and *IFI27*^lo^ PLWH iNs can be associated with biological parameters indicative of disease state or donor characteristics, we analyzed possible associations with the age, duration of HIV-1 infection, NCI, and CD4^+^ T cell count of the respective PLWH donors. Here, we observed a significant association of CD4^+^ T cell count on the day of skin biopsy with *IFI27* expression in iNs (Figure 5G). There was no effect of *IFI27* expression on either age or estimated duration of HIV-1 infection in our cohort (Supplemental Figure 9, B and C). There was a slight trend toward higher GDS in the *IFI27*^lo^ group (Supplemental Figure 9D), but when analyzing whether iN *IFI27* expression negatively correlates with GDS in PLWH, we did not observe a significant effect (Supplemental Figure 9E).

We conclude that postmortem sampling of brain tissue from PLWH confirms that the elevated *IFI27* expression levels we observed in PLWH-derived iNs also occur in the brains of PLWH. Further, the identified DEGs between *IFI27*^hi^ and *IFI27*^lo^ PLWH iNs indicate that distinct mechanisms responsible for type I and II NCI are conserved in iNs and thus support iNs as a suitable model system to study cognitive decline in PLWH.

Overall, differential gene expression analysis on participant-derived iNs revealed 29 DEGs between PLWH- and PWOH-derived iNs potentially revealing additional mechanisms and supporting previous concepts of HIV-1–related neuroinflammation and neurocognitive decline.

## Discussion

We identified an iN gene signature of HIV-1 comprising 29 DEGs including genes associated with neuronal functions and implicated in cognitive decline.

The performed transdifferentiation to generate iNs resulted in around 90% cells expressing pan-neuronal marker gene *TUBB3* (TUJ1). This is in line with the rate obtained by the research group that originally established this protocol and applied it to the study of AD (14). Further, the same protocol resulted in about 15%–20% cells expressing the glutamatergic neuronal marker *SLC17A7* and about 5% cells expressing the GABAergic neuronal marker *GAD1* in a previous study (13). Consistently, we observed rates of 32.8% *SLC17A7^+^* and 7.7% *GAD1^+^* cells among our iNs, underlining the feasibility and comparability of the published protocol among different labs.

Our scRNA-Seq approach to validate neuronal identity and purity of obtained iN populations showed that cells cluster together in a UMAP plot mainly based on origin, most likely based on donor-specific effects. Since UMAPs reveal donor-specific effects based on factors such as age or sex, and we purposefully used cells from a 27-year-old male donor and a 66-year-old female donor for this analysis, these findings further underline that the transdifferentiation protocol retained donor-specific characteristics (75).

One major advantage of the iN model is that these neurons retain the biological age of their donors, as previous studies demonstrated via epigenetic clock analysis following DNA isolation (13, 14, 16, 17). We demonstrate that transcriptomic and stochastic clock approaches also support biological age retention in iNs. Using both the RNAAgeCalc package (24) and a recently published stochastic clock analysis (25), we show that iNs retain donor-specific aging signatures at the transcriptomic level, complementing previous epigenetic evidence.

Subsequent transcriptomic analysis of participant-derived iNs revealed 29 DEGs between virologically suppressed PLWH and PWOH. Although this number appears low compared with work applying the iN system to the study of AD, which found more than 700 DEGs in iNs derived from participants with AD compared with controls, it aligns with a postmortem study conducting microarray-based transcriptome analysis on postmortem brain samples from PLWH and PWOH (8, 14). In that study, approximately 90 probes were significantly regulated in PLWH with no or mild NCI compared with PWOH while analyzing gross brain tissue (not only neurons) across the neostriatum, neocortex, and white matter (8).

Interestingly, the authors found the majority of DEGs in the neostriatum (>80 regulated probes) and less than 10 in neocortex and white matter, respectively (8). This is supported by recent work demonstrating that the brain is a large, compartmentalized organ where neuronal subtype-specific adaptations must be expected (76). As a result, our identified DEGs must be interpreted within the context of the applied iN protocol. As mentioned above, we observed generation of putative glutamatergic and GABAergic neurons and therefore cannot conclude whether effects occur in cholinergic or serotonergic signaling. Thus, future work applying different iN protocols to cohorts of virologically suppressed PLWH and PWOH is needed to identify putative DEGs in distinct neuronal subtypes.

The negative correlation between *FOXL2NB-FOXL2-LINC01391* expression and NCI in PLWH represents a potentially important finding. While we were unable to assess this correlation in PWOH due to limited neurocognitive testing in controls, the biological relationship between expression of this genomic locus and cognitive function suggests a potential pathway in neurocognitive decline. However, neurocognitive assessment in PLWH may be subject to methodological limitations that could overestimate impairment rates (77). The HIV-Cognition Working Group has presented a framework for classification of HIV-related NCI that addresses the potential for overestimation as an important methodological consideration (77, 78). While our study employed established, validated instruments with appropriate demographic corrections, our neurocognitive findings should be interpreted within this evolving methodological context, and the observed correlation between *FOXL2NB-FOXL2-LINC01391* expression and cognitive function warrants validation in future studies using refined assessment approaches. The coordinated downregulation of these 3 genes, controlled by shared regulatory elements on chromosome 3, indicates a common mechanism affecting neuronal function in HIV infection. Future studies with neurocognitive assessment in both PLWH and controls will be necessary to validate this relationship and determine whether expression levels of *FOXL2NB*, *FOXL2*, and *LINC01391* could serve as biomarkers for characterizing neurocognitive decline in HIV.

Finally, we observed increased expression of the inflammatory gene *IFI27* in iNs from PLWH compared with PWOH, consistent with previously conducted postmortem studies (8, 68, 73). Interestingly, *IFI27* has been associated with HIV-1 in several recent studies (79–82). Mackelprang et al. found *IFI27* upregulated in blood samples of PLWH during acute infection, with persistent upregulation in the chronic state (79). The authors concluded that persistent elevation of a narrow set of interferon-stimulated genes, including *IFI27*, underlies chronic immune activation during HIV-1 infection (79). In this regard, our results suggest neuron-derived *IFI27* contributes to this phenomenon. Liu et al. identified genes associated with immunological non-responders to HIV-1 infection in blood samples and found that *IFI27* expression negatively correlated with CD4^+^ T cell count in PLWH (81). Moreover, *IFI27* expression levels significantly predicted poor immune recovery in PLWH, suggesting *IFI27* as a potential biomarker for CD4^+^ T cell recovery. In our cohort, *IFI27* expression in PLWH iNs was likewise negatively associated with CD4^+^ T cell counts, supporting their findings in the context of neurons. In another study, Huang et al. suggested *IFI27* as a potential therapeutic target for HIV infection based on differential expression and PPI network analysis using publicly available data from PLWH and controls (82). Together, our findings in PLWH-derived iNs support this recent association of *IFI27* with HIV-1 and suggest that its expression may also play a role in HIV-1–related NCI.

Besides *IFI27*, none of the other DEGs revealed here has been recognized in the few studies comparing differential gene expression in PLWH- and PWOH-derived postmortem brain tissue (8, 83, 84). This is perhaps not surprising as our findings, focused on the comparison of iNs including a subset of glutamatergic and GABAergic neurons from PLWH and PWOH, have no direct comparator in the literature. In addition, we performed unbiased whole-transcriptome bulk RNA sequencing whereas prior postmortem brain tissue studies, including those identifying *IFI27* as upregulated in PLWH-derived samples, typically conducted targeted gene expression profiling on a subset of previously selected genes (e.g., inflammatory genes) (8, 68, 73). Furthermore, our inclusion of PLWH carefully screened to exclude those with significant neuropsychiatric confounders increases the sensitivity for detecting HIV-1–related effects. While it remains unclear how well iNs represent in vivo molecular mechanisms of NCI in PLWH, our study provides the important evidence that PLWH-derived iNs recapitulate the increased *IFI27* expression observed in postmortem brain tissue, suggesting the iN model captures relevant pathophysiological changes.

Our study has several strengths, including implementing the iN system in the context of infectious disease, comprehensive neurocognitive testing of well-characterized PLWH, and unbiased, whole-genome expression analysis. However, several limitations exist. One limitation was the small cohort size. We believe comparing 6 PLWH iN samples with 7 PWOH iNs was sufficient to address whether gene expression of iNs differentiates virologically suppressed PLWH from PWOH in this proof-of-principle study. This cohort size is consistent with previous postmortem brain studies in this context, which applied similar or smaller cohorts (8, 9, 84, 85). Moreover, our PLWH and PWOH groups are well matched, and PLWH participants underwent extensive neurocognitive assessment and evaluation of HIV-1–related clinical parameters, enabling revealing correlation analyses between iN gene expression and neurocognitive capacities. Nevertheless, the cohort size constrains generalizability. Subsequent studies must validate the DEGs revealed here and provide additional insights into differential gene expression through increased statistical power with larger cohorts. Beyond differential gene expression analysis, we and others must investigate functional differences between PLWH and PWOH iNs, as this model offers the opportunity to study molecular mechanisms of cognitive decline in vitro and explore therapeutic interventions as shown in AD (13, 86). Another limitation was the high number of DEGs between UNA fibroblasts from PLWH and PWOH. Our data indicate that HIV-1 infection with concurrent antiretroviral therapy profoundly affects fibroblast gene expression. Although this did not affect transdifferentiation efficiency in our study, we cannot exclude effects on cellular reprogramming dynamics. This observation is notable because a previous AD iN study found no DEGs between donor-derived fibroblasts from patients and controls (14). As a result, the possibility that HIV-1 infection in the context of suppressive cART may affect cellular reprogramming should be considered in future studies.

Taken together, our study demonstrates that iN gene expression differentiates virologically suppressed PLWH from PWOH, thus identifying an iN gene signature of HIV and revealing potential mechanisms of NCI in PLWH. These findings highlight the utility of participant-derived iNs for studying neuronal gene expression in the context of chronic infectious disease and provide a foundation for future studies to validate the individual genes identified here and expand investigations using different neuronal subtype iN protocols and functional assays.

## Methods

### Sex as a biological variable.

Female and male participants were enrolled in this study. Sex was not specifically analyzed as a biological variable because of the limited cohort size.

### Statistics.

Statistical significance for bulk RNA-sequencing experiments was tested using the Wald test in DESeq2 (*P* value) and corrected for FDR using the Benjamini-Hochberg as described in the respective Supplemental Methods and figure legends. Statistical significance for publicly available gene array data sets was tested with unpaired, 2-sided *t* tests between conditions. *P* values or adjusted *P* values less than 0.05 (and for bulk RNA-Seq analysis when log2fc > ± 0.5) were considered significant.

### Study approval.

The study was approved by the Rockefeller University Institutional Review Board (IRB) and acknowledged by the Weill Cornell Medicine (WCM) IRB. Written informed consent was obtained from all participants prior to their entering the study.

### Data availability.

Raw RNA-Seq and scRNA-Seq data have been deposited in the NCBI Sequence Read Archive (SRA) under BioProject PRJNA1353888 and are publicly accessible. Complete lists of differentially expressed genes are available as Supplemental Tables 2, 3, and 8. The code for the stochastic data-based clock can be found at GitHub (https://github.com/Meyer-DH/StochasticAgingClock; commit ID caf31fd), and the code as well as the RNAAgeCalc package to conduct the transcriptomic age analysis can be found at Bioconductor (https://bioconductor.org/packages/release/bioc/html/RNAAgeCalc.html). Values for all data points shown in graphs are provided in the Supporting Data Values file in the supplement. The complete Methods section is available online in the Supplemental Methods.

## Author contributions

THE performed conceptualization. PNO, YW, SMM, BAS, DHM, DFN, MS, and THE developed methodology. PNO, YW, SAB, MS, MAS, SMM, BAS, DHM, LASB, SMM, AH, and THE performed investigation. PNO, SAB, AH, SMM, BAS, and DHM performed visualization. LCN, MY, DFN, TZ, and THE performed funding acquisition. TZ and THE performed project administration. RBJ, DFN, MY, LCN, TZ, and THE supervised. PNO performed writing – original draft. PNO, YW, SAB, SMM, MAS, DHM, AH, BAS, MS, KR, LASB, DB, RBJ, MY, DFN, LCN, TZ, and THE performed writing – review and editing.

## Funding support

This work is the result of NIH funding, in whole or in part, and is subject to the NIH Public Access Policy. Through acceptance of this federal funding, the NIH has been given a right to make the work publicly available in PubMed Central.

- National Institute on Aging (NIA) grant R21AG071433 (THE).
- National Institute of Neurological Disorders and Stroke (NINDS) grant R21NS126094 (THE).
- German Research Foundation (DFG) grant HU 1636/13-1 (PNO).
- NIA grant R56AG078970 (DFN).
- NINDS grant R01NS117458 (LCN).
- National Institute of Allergy and Infectious Diseases (NIAID) grant UM1AI164559 (LCN).
- National Institute of Mental Health (NIMH) grant R01MH130197 (LCN).
- National Institute on Drug Abuse (NIDA) grant U01DA058527 (LCN).
- NIDA grant R01DA052027 (LCN).
- NIAID grant R56AI125128 (MY).
- Koeln Fortune grant 85/2025 from Faculty of Medicine of University of Cologne (DHM).

## Supplementary Material

Supplemental data

Supplemental table 1

Supplemental table 2

Supplemental table 3

Supplemental table 4

Supplemental table 5

Supplemental table 6

Supplemental table 7

Supplemental table 8

Supporting data values

## Version 1 12/01/2025

In-Press Preview

## Version 2 01/23/2026

Electronic publication
