# Supplementary material for: Transcriptional signature of induced neurons differentiates virologically suppressed people with HIV from people without HIV
Source: JCI Insight. 2025 Nov 25;11(2):e190445. doi: 10.1172/jci.insight.190445 (PMC12892921; doi:10.1172/jci.insight.190445)
Supplement: Supplemental data [file jciinsight-11-190445-s261.pdf]

Supplements

Transcriptional signature of induced neurons differentiates virologically suppressed people with HIV from people without HIV

Philipp N. Ostermann[1#], Youjun Wu[2*], Scott A. Bowler[1*], Samuel Martínez-Meza[1,3], Mohammad Adnan Siddiqui[4], David Meyer[5,6], Alberto Herrera[1], Brandon A. Sealy[3], Mega Sidharta[2], Kiran Ramnarine[2], Leslie Ann St. Bernard[1], Desiree Byrd[7,8], R. Brad Jones[1], Masahiro Yamashita[4], Douglas F. Nixon[1,3], Lishomwa C. Ndhlovu[1], Ting Zhou[2], Teresa H. Evering[1]**

[1]Division of Infectious Diseases, Department of Medicine, Weill Cornell Medicine, New York, NY 10065, USA

[2]The SKI Stem Cell Research Facility, The Center for Stem Cell Biology and Developmental Biology Program, Sloan Kettering Institute, New York, NY 10065, USA

[3]Institute of Translational Research, Feinstein Institutes for Medical Research, Northwell Health, Manhasset, NY 11030, USA

[4]Aaron Diamond AIDS Research Center, Columbia University Vagelos College of Physicians and Surgeons, New York, NY 10032, USA

[5]Institute for Genome Stability in Aging and Disease, University Hospital and University of Cologne, 50923 Cologne, Germany

[6]Cologne Excellence Cluster for Cellular Stress Responses in Aging-Associated Diseases (CECAD), Center for Molecular Medicine Cologne (CMMC), University of Cologne, 50923 Cologne, Germany

[7]Department of Neurology, The Icahn School of Medicine at Mount Sinai, New York, NY, USA

[8]Department of Psychology, Queens College and the Graduate Center, City University of New York, New York, NY, USA

[#]Current address: Translational Pain Research, Department of Anaesthesiology and Intensive Care Medicine, Faculty of Medicine and University Hospital Cologne, University of Cologne, 50931 Cologne, Germany

*These authors contributed equally

**Correspondence: evering@med.cornell.edu (T. H. Evering)

Methods

Sex as a biological variable

Female and male participants were enrolled in this study. Sex was not specifically analyzed as a biological variable due to the limited cohort size.

Study participants

Enrolled participants underwent a medical history, physical and neurological examination and psychiatric and substance use history at the screening visit. For participants screened at our site (all 6 PLWH and 2 PWOH), exclusion criteria adapted from Rippeth et al. (1) included severe neurological or Diagnostic and Statistical Manual Fourth Edition-Text Revision (DSM-IV-TR) (2) psychiatric illness that affects cognitive functioning (e.g. schizophrenia, bipolar affective disorder), current diagnosis of major depressive disorder as assessed by the Patient Health Questionnaire-9 (PHQ-9) item depression scale (3) and not on stable antidepressant medication greater than 30 days, a history of head injury with loss of consciousness more than 30 min, DSM-IV-TR diagnostic criteria for alcohol or illicit substance abuse or dependence, not in remission, within 1 year of the screening visit (excluding marijuana), moderate or higher efavirenz attributable central nervous system (CNS)-related toxicity or serologic evidence of untreated syphilis or positive hepatitis C serology. All PLWH had documented treatment for at least 1 year with cART and plasma HIV-1 RNA levels below 50 copies/ml for a minimum of 6 months prior to study entry. Regarding the repository samples, the 6 donors for PWOH control fibroblasts were classified as either "apparently healthy individuals" (NIGMS Human Genetic Cell Repository) or documented as "Affected: No" (NIA Aging Cell Culture Repository), indicating exclusion of major known medical conditions.

Neuropsychological evaluation

The neuropsychological evaluation of the six PLWH recruited at the Rockefeller University was performed as described previously (4). Comprehensive neuropsychological evaluation assessing seven cognitive domains associated with HAND (attention/working memory; processing speed;

learning; recall; abstraction/executive functioning; verbal fluency; and motor skills) was adapted from Rippeth et al. (1) and performed at the study visit by a trained study psychometrist. All tests were administered following standardized protocols in a controlled testing environment to ensure data quality.

The neuropsychological test battery assessed verbal fluency using FAS letter fluency and category fluency (animals). Processing speed was evaluated through the WAIS-III digit symbol, WAIS-III symbol search, and Trail Making Test Part A. We assessed attention and working memory using the Paced Auditory Serial Addition Task (PASAT) and WAIS-III letter-number sequencing. Learning was measured with the Hopkins Verbal Learning Test-Revised (HVLT-R) Trials 1-3 and Brief Visuospatial Memory Test-Revised (BVMT-R) Trials 1-3. Recall was assessed using the HVLT-R delayed recall. For abstraction and executive functioning, we administered the Wisconsin Card Sorting Test-64 item, Trail Making Test Part B, and Stroop color and word interference test. Motor skills were evaluated using the Grooved Pegboard test (dominant and non-dominant hands). Additionally, participants completed the Wide Range Achievement Test 3 (WRAT3) reading and spelling subtests to provide an assessment of premorbid intellectual functioning.

Using methods that correct for age, education, sex and ethnicity where appropriate, raw scores for all tests were transformed into T-scores (1). T-scores were then converted to deficit scores, which range from a minimum of 0 in the case of no impairment, to a maximum of 5 (5, 6). Calculating the sum of all deficit scores in the testing battery and then dividing by the number of administered tests allowed for determination of the global deficit score (GDS) for each participant, which provides a continuous measure of neurocognitive impairment (NCI). Individuals were classified as neurocognitively impaired if GDS scores were ≥ 0.50, a cutpoint that has been demonstrated as highly sensitive and specific for the classification of HAND (5, 6).

Dermal fibroblast isolation and propagation

The majority of skin fibroblast cultures were generated from skin punch biopsies specifically for this study (Table S1). Skin samples from all six PLWH and two of eight PWOH as detailed in the

manuscript were collected via skin punch biopsy under Rockefeller University IRB-approved protocol. Dermal fibroblasts were isolated from skin biopsy samples and expanded by the MSK Stem Cell Research Facility. Briefly, a 6 mm diameter skin biopsy was dissected into 10 - 15 smaller pieces, which were then plated on a 10 cm dish. Two to three pieces of samples were transferred into each well of a 6-well plate coated with 0.1 % gelatin and containing 500 µl fibroblast culture medium. The culture medium consisted of DMEM high glucose (ThermoFisher) supplemented with 10 % fetal bovine serum (HyClone), 1X NEAA (ThermoFisher), and 1X L-glutamine (ThermoFisher). A circular coverslip (FisherScientific) was carefully placed on top of the biopsy samples, and 1.5 ml of fibroblast culture medium was added onto the coverslip. Fibroblasts were observed approximately two weeks after plating and were passaged after three weeks onto gelatin-coated plates using trypsin-EDTA (0.05 % EDTA) for expansion. Dermal fibroblasts from six of eight PWOH control participants, chosen to match the demographics of the PLWH, were obtained via MTA from the *Coriell Institute for Medical Research* (NJ, USA).

Generation of induced neurons

The performed protocol to generate induced neurons (iNs) was adapted from (7, 8). For the generation of lentiviral vectors, six million HEK293T cells (ATCC; CRL-3216) were seeded into a 0.1 % gelatin-coated T150 cell culture flask in DMEM (Gibco) supplemented with 10 % fetal calf serum (FCS). The next day, cells were transfected with 6 µg of the packaging plasmid *psPAX2* (psPAX2 was a gift from Didier Trono (School of Life Sciences, École Polytechnique Fédérale de Lausanne, Lausanne 1015, Switzerland); Addgene plasmid # 12260 ; http://n2t.net/addgene:12260 ; RRID:Addgene_12260), 6 µg of the envelope plasmid *pMD2.G* (pMD2.G was a gift from Didier Trono (Addgene plasmid # 12259 ; http://n2t.net/addgene:12259 ; RRID:Addgene_12259), and 6 µg of the transfer plasmid *pLVX-UbC-rtTA-Ngn2:2A:Ascl1* (pLVX-UbC-rtTA-Ngn2:2A:Ascl1 was a gift from Fred Gage (Laboratory of Genetics, The Salk Institute for Biological Studies, La Jolla, United States); Addgene plasmid # 127289 ; http://n2t.net/addgene:127289 ; RRID:Addgene_127289) (9). The transfection mix containing

polyethylenimine (60 µg/ml) (Polysciences) in 1 ml DMEM, and the three plasmids was added to the cells after an incubation period of 30 min. At 24 h post-transfection, the culture medium was exchanged to 15 ml fresh DMEM supplemented with 10 % FCS. At 48 h post-transfection, lentiviral vectors were harvested by pelleting any cells and cell debris at 400 x *g* for 5 min, aliquoting the resulting supernatant and storing at -80°C. UNA fibroblasts were generated by transducing 500,000 dermal fibroblasts with 500 µl of the lentiviral vector stock in a T25 cell culture flask while adding 4 µg/ml polybrene (Tocris) to improve transduction efficiency. At 24 h post-transduction, the medium was exchanged to fresh fibroblast medium. Selection with puromycin (1 µg/ml) (Sigma) started 72 h post-transduction. Transduced and selected fibroblasts (UNA fibroblasts) were passaged once and then frozen in liquid nitrogen. To generate iNs, all UNA fibroblasts were thawed the same day and processed in parallel to reduce batch effects during RNA sequencing. UNA fibroblasts were passaged several times after thawing and the neuronal conversion was performed as previously described (8). At day 21 of neuronal conversion, successfully converted iNs were isolated by FACS via staining for the neuronal marker PSA-NCAM. For this, cells were detached from the cell culture flasks with trypsin-EDTA and collected in FACS buffer consisting of 5 % FCS in PBS. Cells were pelleted (400 x *g*, 5 min) and incubated in a total of 200 µl FACS buffer containing the PE-conjugated PSA-NCAM antibody (Miltenyi Biotec; clone 2-2B; cat. no. 130-117-394) at a 1:100 dilution. After an incubation for 30 min at 4°C in the dark, the cells were washed twice with 500 µl FACS buffer and resuspended in 300 µl FACS buffer containing 1x DAPI (Thermo Scientific) used as live/dead stain. Cell sorting was performed on a BD FACSymphony™ S6 Cell Sorter at the WCM CLC Flow Cytometry Core Facility. Sorted cells were pelleted and then either lysed according to the respective downstream protocol or cultured in BrainPhys medium (StemCell) supplemented with B27 (1x) (Thermo), N2 (1 %) (StemCell), GDNF (20 ng/ml) (StemCell), BDNF (20 ng/ml) (StemCell), db-cAMP (500 µg/ml) (StemCell), and Laminin (1 µg/ml) (Thermo). For the first 24 hours post-FACS, we supplemented the medium with 10 µM ROCK inhibitor (MedChemExpress).

Microscopic analysis and immunocytochemistry

Medium was removed, and the cells incubated in 4 % PFA for 20 min at room temperature (RT). After washing with PBS, cells were permeabilized with 0.1 % Triton-X-100 in PBS for 10 min at RT. After two additional washing steps with PBS, a blocking solution (2 % BSA in PBS) was applied for 1 h at RT. Primary antibodies were diluted in blocking solution and the cells were incubated with this antibody solution overnight at 4°C. Cells were washed twice with PBS and incubated with the secondary antibodies and Hoechst to stain nuclei for 2 hours at RT in the dark. Microscopic images were taken with the Olympus IX81 microscope (Olympus) using the *Slidebook* (version 6) software (3i). Image analysis has been performed with Fiji (10). The following antibodies were used in this study: Mouse-anti-Tubulin beta-3 (TUBB3) antibody (BioLegend; clone TUJ1; cat. no. 801201, previously MMS-435P), chicken-anti-MAP2 antibody (ThermoFisher; cat. no. PA1-10005), Alexa 488-conjugated goat anti-mouse IgG (ThermoFisher; cat. no. A28175) and Alexa 568-conjugated anti-chicken IgY (ThermoFisher; cat. no. A78950).

Multi-electrode array analysis

Multi-electrode array (MEA) 48-well plates containing 16 electrodes per well (M768-tMEA48B, Axion Biosystems) were pre-treated with 80 µl 0.01% Poly-L-ornithine (Sigma) and incubated at 37°C and 5% CO_2 for one hour. Plates were washed three times with 200 µl of deionized water and left to dry overnight in a biosafety cabinet with the lids slightly ajar. The next day, 5 µl of laminin (Sigma) at 1 µg/ml was applied to the center of each well covering the microelectrode area and plates were incubated at 37°C and 5% CO_2 for two hours. Laminin was removed by washing three times with 200 µl of neural conversion (NC) medium leaving 200 µl of medium in each well to prevent drying. Cells were dissociated using trypsin and seeded as 5 µl droplets into the center of each well (approx. 100,000 cells/well. Plates were incubated at 37°C and 5% CO_2 for 2-3 hours to allow cell attachment and 250 µl of NC medium was gently added to each well. NC medium was switched to maturation media (supplemented BrainPhys media, as previously described) on day 4 on MEA plates and cells were cultured for another 5 days. A stimulation

protocol (electrical pulses of 500 mV for 100 µs; every 40 seconds on all 16 electrodes) was applied to analyze spontaneous and evoked electrical activity using the Maestro Pro MEA platform (Axion Biosystems, USA) together with the AxIS Navigator software (Axion Biosystems).

Single-cell RNA sequencing and analysis

Cells were pelleted after FACS by centrifugation at 1,200 x *g* for 10 min. Medium was removed until only about 1 ml was left on top of the cells. The cells were resuspended and transferred into 1.5 ml tubes. After another centrifugation step (1,200 x *g* for 5 min), the complete supernatant was removed, and cells resuspended in 50 µl PBS containing 0.04 % BSA. Single-cell (sc)RNA sequencing has been performed at the Genomics Resources Core Facility (GRCF) at Weill Cornell Medicine. In brief, the 10X Libraries were sequenced on the Illumina NovaSeq6000 platform with pair-end reads (28 bp for read 1 and 90 bp for read 2). Sequencing data were analyzed by the 10X Cell Ranger pipeline (v7.1.0) in two steps. In the first step, Cell Ranger mkfastq demultiplexed samples and generated FASTQ files and in the second step, Cell Ranger count aligned FASTQ files to the 10X pre-built human reference genome (refdata-gex-GRCh38-2020-A) with standard parameters as described on 10X Genomics (https://www.10xgenomics.com/support/software/cell-ranger/latest/analysis/running-pipelines/cr-gex-count) and extracted gene expression UMI counts matrix. Count matrices were processed in RStudio using the *Seurat* package (11-13). Cells were filtered as previously described (>300/<10,000 unique feature counts and < 30 % mitochondrial reads), which resulted in 5994 (Participant 4642) and 8368 cells (Participant 3962) for downstream analysis (7). Data was normalized using the global-scaling normalization method with *LogNormalize* (scale factor 10,000). A subset of 2,000 features with high cell-to-cell variation was identified and scaled for downstream analysis. UMAP plots were generated using the identified dimensionality during principal component analysis. Percentages of cells expressing certain genes were determined using the *scCustomize* package (14).

Bulk-RNA sequencing and analysis

Total RNA was extracted using the RNeasy kit (Qiagen) including the 15 min on-column DNase treatment. RNA integrity and quantity has been determined using the TapeStation instrument (Agilent). All RNA samples exhibited an RNA integrity number (RIN) above 9.0. Libraries were sequenced with paired-end 50 bps on a NovaSeqXplus sequencer. Raw sequencing reads in BCL format were processed through bcl2fastq 2.20 (Illumina) for FASTQ conversion and demultiplexing. After trimming the adaptors with cutadapt (1.18), RNA reads were aligned and mapped to the GRCh38 human reference genome by STAR (2.5.2) and transcriptome reconstruction was performed with Cufflinks (2.1.1) (15, 16). Raw read counts per gene were extracted using HTSeq-count v0.11.2 (17). Read count matrices were imported into RStudio and differential gene expression analysis was performed using the *DESeq2* package (18). Low count genes (>10 reads) were pre-filtered and effect sizes were shrinked for visualization in MA plots using the *apeglm* method (19). Statistical significance was tested via the Wald test (p-value) implemented in *DESeq2* and corrected for false discovery rates (FDR) using the Benjamini-Hochberg method (p-adj.). DESeq2 median ratios count normalization was used. For visualization and cluster analysis, count data transformation was performed using the *vst* function (20). Gene set enrichment analysis was conducted with *EnrichR*, which uses Fisher's exact test or the hypergeometric test (p-value) and FDR correction via the Benjamini-Hochberg method (p-adj.) (21-23), and the free GSEA 4.3.3 software (24, 25). Gene sets used for this study were derived from Gene Ontology (26, 27), Jensen DISEASES (28), SynGO (29), Reactome (30, 31), DisGeNet (32), and Molecular Signatures (MSigDB) hallmark gene set databases (33).

Transcriptomic clock analysis

RNA-seq quantification for clock predictions was performed using the human reference genome hg38, with transcript annotation based on the GENCODE v39 (GRCh38) GTF file, downloaded via the GTEx reference resource (https://www.gtexportal.org/home/downloads/adult-gtex/reference). A decoy-aware transcriptome index was generated using Salmon (v1.9) (34) with

k-mer size set to -k 31. Transcript quantification was conducted with Salmon quant using the following parameters: --validateMappings, --gcBias, and --seqBias. Predictions were obtained for both in-house data (UNA-fibroblasts and iNs) and healthy donor-derived fibroblast, iNs and iPSC transcriptomes from Mertens et al. (2015) (EMBL-EBI ArrayExpress: E-MTAB-3037) (35). To estimate transcriptomic biological age, we employed two independent strategies: (1) a panel of 16 published clocks, and (2) an ensemble of 100 stochastic clocks trained on simulated data with accumulating stochastic variation (see below).

RNAAgeCalc Clock Analysis

We used the RNAAgeCalc package (36) for eight different clock predictions (DESeq2, Pearson, Dev, deMagalhaes, GenAge, GTExAge, Peters, and all), each combined with two stratification settings (stype = caucasian and stype = all), using the alltissues parameter.

Stochastic Clock Analysis

Stochastic clocks were trained using as previously published (37), based on accumulating stochastic variation. Briefly, all RNA-seq samples (including public datasets) were pre-processed using edgeR (38) (TMM normalization and log-transformed CPM), after which the samples were min-max normalized independently. Starting from the youngest normalized sample (ERR668347 (35)), we iteratively added normally distributed stochastic variation ($\sigma = 0.01$) in 89 steps to simulate transcriptomic aging. The simulated training data and independent biological test data were binarized as described previously (39). An ElasticNet regression model (alpha=1, l1_ratio=0.1) was trained on the simulated dataset to predict how often stochastic variation was added to the biological starting point. To ensure robustness we simulated 100 clocks, each independently trained using different random seeds (0–99).

Cross-Clock Analysis and Mixed Model Evaluation

To enable joint analysis, each predicted age value (from the 16 RNAAgeCalc clocks or 100 stochastic clocks) was min-max normalized to the range [0, 1] per clock. We used a linear mixed model using the statsmodels Python package (smf.mixedlm) (40) to test whether the predicted age shows distinct slopes across cell types. The model formula was 'predicted_age ~ chronological_age * cell_type', with additional variance components modeled using 0 + C(variable), where variable denotes clock identity. Repeated measures within samples (i.e., multiple predictions per individual) were accounted for via the groups parameter (set to individual ID).

Protein-protein interaction (PPI) network analysis

We determined 1st-order interaction partners using the free open-source IntAct Molecular Interaction Database system (EMBL-EBI) (41), and the human reference interactome (HuRI) map (Center for Cancer Systems Biology at Dana-Farber Cancer Institute) (42). We generated and retrieved lists of the 1st-order interaction partners of the here identified DEGs based on the underlying literature curation and direct user submission (IntAct) as well as the unbiased, systematic, yeast two-hybrid screen for PPIs (HuRI).

Statistical analysis and software

Statistical analysis in the context of our scRNA and bulk-RNA sequencing experiments has been described in the respective Supplemental Methods and figure legends. P-values or adjusted p-values less than 0.05 were considered significant. Genes were considered significantly differentially expressed when p-adj. < 0.05 and log2fc > +/- 0.5. To analyze publicly available gene array data, we obtained respective data sets on *IFI27* expression values in post-mortem brain tissue from the curated transcriptome dataset collection Gene Expression Browser [http://hiv.gxbsidra.org/dm3/geneBrowser/list] (43). Comprehensive information about the three downloaded data sets is provided in Table S7. Statistical significance was tested with unpaired, two-sided *t*-test between conditions. Besides the aforementioned software, *GraphPad* was used

for a subset of statistical analysis and plots. *Inkscape* was used for illustrations and finalization of figures. *BioRender* was used to generate a subset of schemes. Statistical analyses were run with either *RStudio* using the *DESeq2* or *Seurat* package, *GraphPad*, or *EnrichR* and has been depicted throughout the manuscript where applied.

Study approval

The study was approved by the Rockefeller University Institutional Review Board (IRB) and acknowledged by the Weill Cornell Medicine IRB. Written informed consent was obtained from all participants prior to their entering the study.

Data availability

Please contact the corresponding author Teresa H. Evering (evering@med.cornell.edu) for any inquiries regarding the used material and uploaded data. Raw RNA-seq and scRNA-seq data have been deposited in the NCBI Sequence Read Archive (SRA) under BioProject PRJNA1353888 and are publicly accessible. Complete lists of differentially expressed genes are available as supplementary material. The code for the stochastic data-based clock can be found at https://github.com/Meyer-DH/StochasticAgingClock and the code as well as the RNAAgeCalc package to conduct the transcriptomic age analysis can be found at https://bioconductor.org/packages/release/bioc/html/RNAAgeCalc.html. Values for all data points shown in graphs are provided in the “Supporting data values” file in the supplements.

Supplementary figures

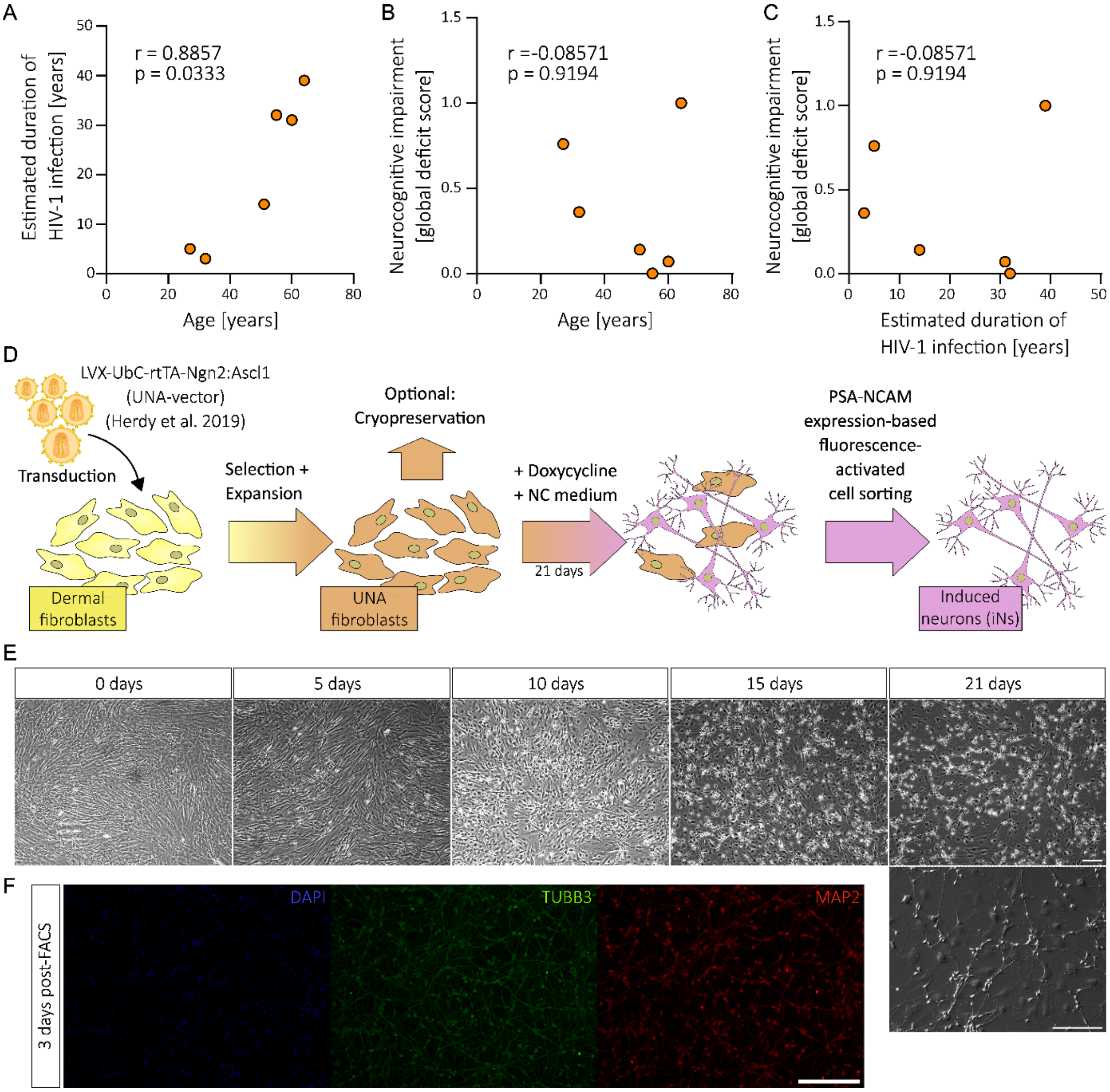

Figure S1 Study cohort and transdifferentiation protocol used to generate induced neurons (A-C) Correlation of age, estimated duration of HIV-1 infection, and neurocognitive impairment in the PLWH study group (n =6). Data points are individual values and correlation analysis performed via two-tailed, nonparametric Spearman. **(D)** Scheme illustrating the workflow of participant-derived iNs generation following the previously published *Mertens* protocol (8). **(E)** Microscopic images of participant-derived skin fibroblasts (donor 100-O3) at different days during the transdifferentiation protocol. **(F)** Single channel microscopic images after immunocytochemistry of induced neurons (iNs) 3 days post-FACS stained for TUBB3 (TUJ1), MAP2 and nuclei (DAPI). **(E-F)** Scale bars are 20 µm.

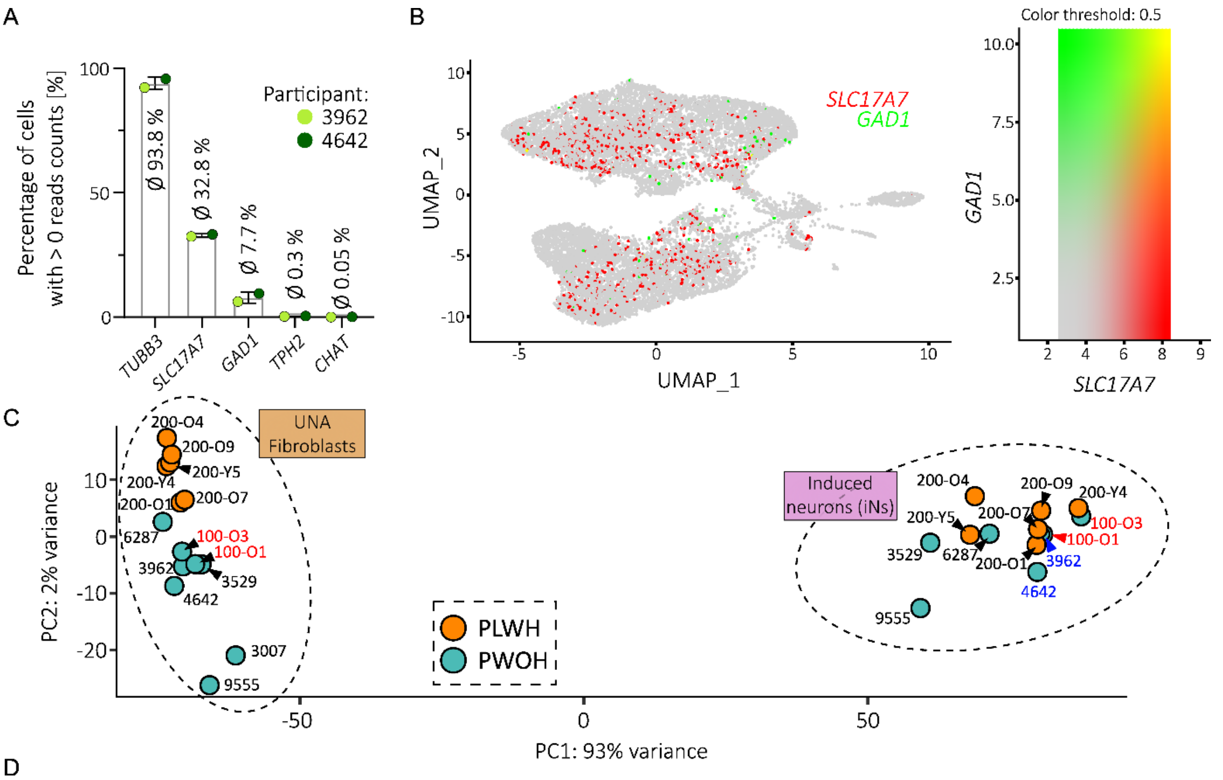

D

↓ Downregulated in iNs: GO Biological Process			
Rank	Name	p-value	adjusted p-value
1	Extracellular Matrix Organisation	1.48e-22	6.10e-19
2	Pos. Regulation of Angiogenesis	2.80e-14	5.16e-11
3	Regulation of Angiogenesis	3.76e-14	5.16e-11
4	Pos. Regulation of Vasculature Development	9.04e-14	9.33e-11
5	Regulation of Cell Migration	1.23e-13	1.01e-10

↓ Downregulated in iNs: GO Cellular Component			
Rank	Name	p-value	adjusted p-value
1	Collagen-Containing Extracellular Matrix	8.94e-49	2.72e-46
2	Cell-Substrate Junction	9.25e-26	1.41e-23
3	Focal Adhesion	6.67e-25	6.75e-23
4	Endoplasmic Reticulum Lumen	4.97e-15	3.78e-13
5	Basement Membrane	3.06e-12	1.87e-10

Figure S2 Neuronal subtype marker gene expression and loss of fibroblast gene expression in induced neurons (A) Percentage of cells from the scRNA analysis performed here that express the annotated pan-neuronal and neuronal subtype marker genes. Data presented as individual data points with mean ± SD. **(B)** UMAP plot showing *SLC17A7* and *GAD1* expression patterns and values among iNs. **(C)** PCA plot showing the calculated distance between bulk-RNA samples with annotations for every single study participant. Bulk-RNA sequencing of iNs was performed after sorting for PSA-NCAM^{+} cells. **(D)** Top 5 ranked gene ontology (GO) terms of Biological Processes and Cellular Components associated with the significantly downregulated genes in iNs compared to UNA fibroblasts.

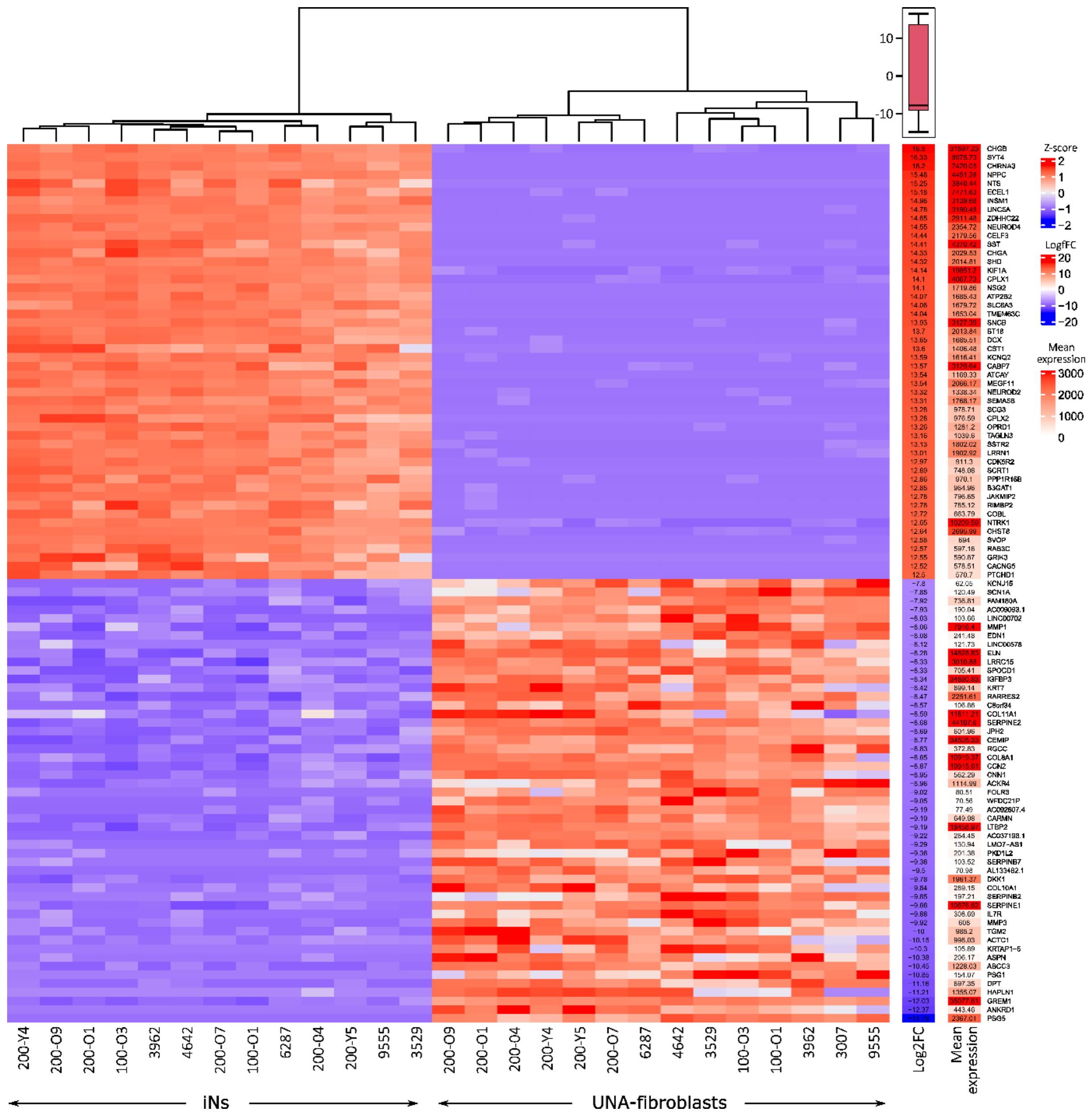

Figure S3 Heatmap showing clustering of UNA fibroblasts and iNs according to the top 50 up- and downregulated genes This heatmap displays the mean expression values of the top 50 up- and downregulated statistically significant (p-adj. < 0.05, log2fc > +/- 0.5) DEGs and the resulting log2 fold change between the UNA fibroblast and iNs samples based on our bulk-RNA analysis.

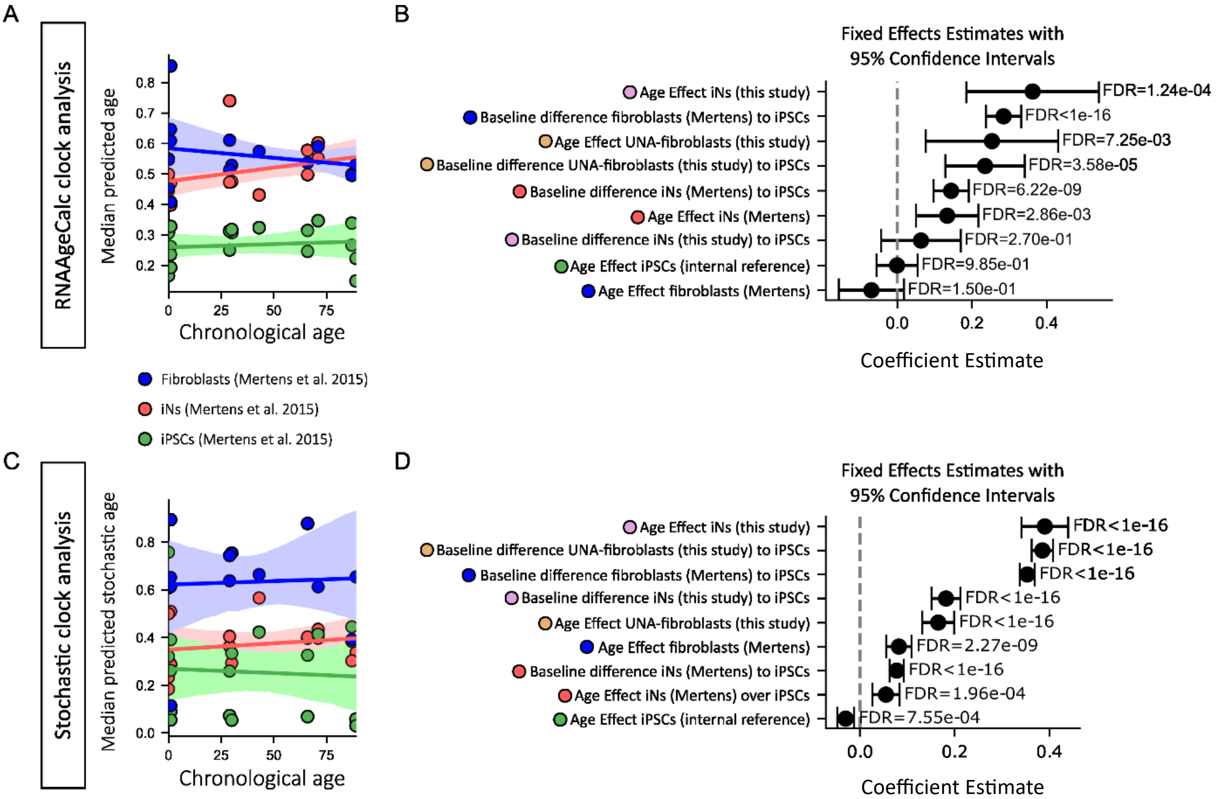

Figure S4 RNAAgeCalc clock and stochastic clock analyses show conservation of donor transcriptomic age in the context of donor-derived iNs but not donor-derived iPSCs (A) Median predicted transcriptomic age (scaled 0 – 1) across 16 RNAAgeCalc clocks plotted against chronological age for donor-derived human iPSCs (green), fibroblasts (blue), and iNs (red). **(B)** Fixed effect estimates from a linear mixed model quantifying baseline differences and age-related slopes across cell types based on RNAAgeCalc predictions using iPSCs as internal reference. Bars represent 95% confidence intervals; FDR-adjusted p-values indicate statistical significance. **(C)** Median predicted transcriptomic age (scaled 0 – 1) of an ensemble of 100 stochastic clocks plotted against chronological age for donor-derived human iPSCs (green), fibroblasts (blue), and iNs (red). **(D)** Fixed effect estimates from a linear mixed model applied to stochastic age predictions using iPSCs as internal reference. Bars represent 95% confidence intervals; FDR-adjusted p-values indicate statistical significance. (**A - D**) Transcriptomic data for the analysis of UNA fibroblasts (“This study”; brown) and iNs (“This study”; pink) was generated during this study, and of fibroblasts (“Mertens”; blue), of iNs (“Mertens”; red), and of iPSCs (“Mertens”; green) as control for age reset was derived from Mertens et al. 2015 (EMBL-EBI ArrayExpress: E-MTAB-3037) using publicly available data on healthy donors.

A

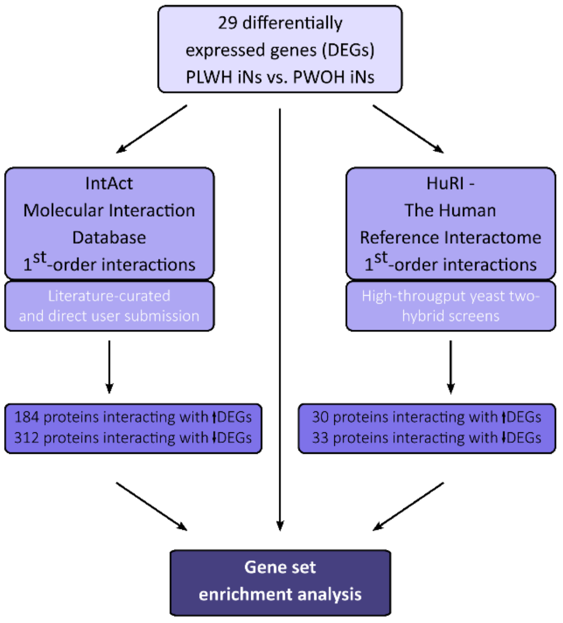

B

Name	p-value	adjusted p-value
**GO Biological Process**		
Regulation Of Apoptotic Process	7.37E-9	1.20E-5
Positive Regulation Of Apoptotic Process	8.99E-9	1.20E-5
Positive Regulation Of Neuron Apoptotic Process	7.61E-7	3.39E-4
Positive Regulation Of Neuron Death	1.07E-6	4.09E-4
Positive Regulation Of Programmed Cell Death	2.03E-6	5.43E-4
Spontaneous Synaptic Transmission	4.69E-6	1.14E-3
**Reactome**		
Assembly Of Collagen Fibrils And Other Multimeric Structures	9.06E-8	4.80E-5
Extracellular Matrix Organization	1.61E-7	5.68E-5
Collagen Formation	2.22E-7	5.70E-5
**SynGO 2024**		
Synaptic Vesicle Endocytosis	5.26E-4	3.63E-2
**Jensen DISEASE**		
Neurodegenerative disease	4.58E-8	2.88E-5
Spinocerebellar ataxia type 1	3.60E-4	3.76E-2
Dementia	5.86E-4	4.21E-2
**DisGeNET**		
Alzheimer's Disease	1.73E-9	8.44E-7
Neurodegenerative Disorders	2.74E-7	6.09E-5
Dementia	2.50E-6	2.48E-4

Figure S5 Protein-protein interaction network mapping supports differential ECM organization, and synaptic transmission in PLWH iNs and indicates neuronal apoptosis as another affected pathway (A) Flowchart summarizing the performed protein-protein interaction (PPI) network mapping approach using the IntAct Molecular Interaction Database (41) and The Human Reference Interactome (HuRI) (42). **(B)** Gene set enrichment analysis-derived terms significantly enriched within the obtained PPI network of 1st-order interaction partners of the 29 DEGs identified here between PLWH- and PWOH-derived iNs.

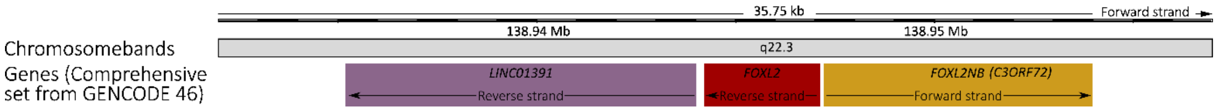

Figure S6 Gene locus of *LINC01391*, *FOXL2*, and *FOXL2NB* The exact location of the three genes *LINC01391*, *FOXL2*, and *FOXL2NB* on human chromosome 3 is shown. Information and original graphic retrieved from the freely available genomic resource Ensembl (https://www.ensembl.org; 9th June 2024, 12:33) (44).

A

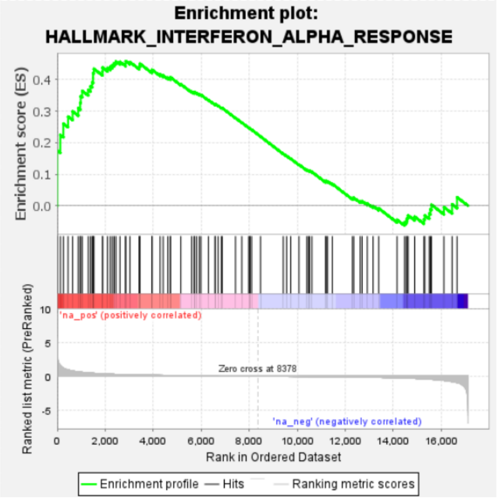

B

SYMBOL	RANK IN GENE LIST	RANK METRIC SCORE	RUNNING ES	CORE ENRICHMENT
IFI27	5	4.7957587242126465	0.17451571	Yes
MX1	130	1.5702553987503052	0.22446814	Yes
IFI44L	257	1.1914876699447632	0.2604967	Yes
IFIT2	453	0.9234875440597534	0.28270283	Yes
IFITM1	642	0.7599382996559143	0.2993587	Yes
IFIT3	875	0.6450046896934509	0.30924022	Yes
IRF7	958	0.6074786186218262	0.32656604	Yes
IFITM3	977	0.600405216217041	0.34739387	Yes
IFIH1	1043	0.5690356492996216	0.3643171	Yes
HERC6	1292	0.4915499687194824	0.36766514	Yes
CMPK2	1388	0.46530136466026306	0.37904474	Yes
DDX60	1456	0.4456027150154114	0.39135125	Yes
LGALS3BP	1494	0.43228933215141296	0.4049349	Yes
CD47	1495	0.4321454167366028	0.420687	Yes
IFI44	1527	0.42368772625923157	0.43430957	Yes
IFITM2	1886	0.34451016783714294	0.42583567	Yes
PARP12	1890	0.3439132869243622	0.43819535	Yes
LAP3	2098	0.30903199315071106	0.4372991	Yes
IRF1	2215	0.29453015327453613	0.44122025	Yes
TRIM21	2301	0.2820138931274414	0.44650635	Yes
DHX58	2374	0.27164226770401	0.45217812	Yes
ISG15	2446	0.2623083293437958	0.4575684	Yes
TRIM5	2616	0.24179725348949432	0.4564538	Yes
PSME1	2848	0.21908099949359894	0.45086882	Yes
IRF9	2851	0.21882934868335724	0.45872784	Yes

Figure S7 Unfiltered gene enrichment analysis shows a significant positive association of the PLWH-derived iNs transcriptome with the hallmark gene set Interferon-α response (A) Enrichment plot of the Hallmark Interferon-α response after analyzing the differential gene expression profile of PLWH-derived iNs when compared to PWOH-derived iNs via unfiltered gene set enrichment analysis (GSEA) of a pre-ranked gene list based on log2FC using the GSEA 4.3.3 software (FDR q-value 0.11; p-value 0.026, NES 1.462). **(B)** List of genes that have contributed to a positive core enrichment during the unfiltered gene set enrichment analysis including several inflammatory response genes previously associated with HIV-1 infection.

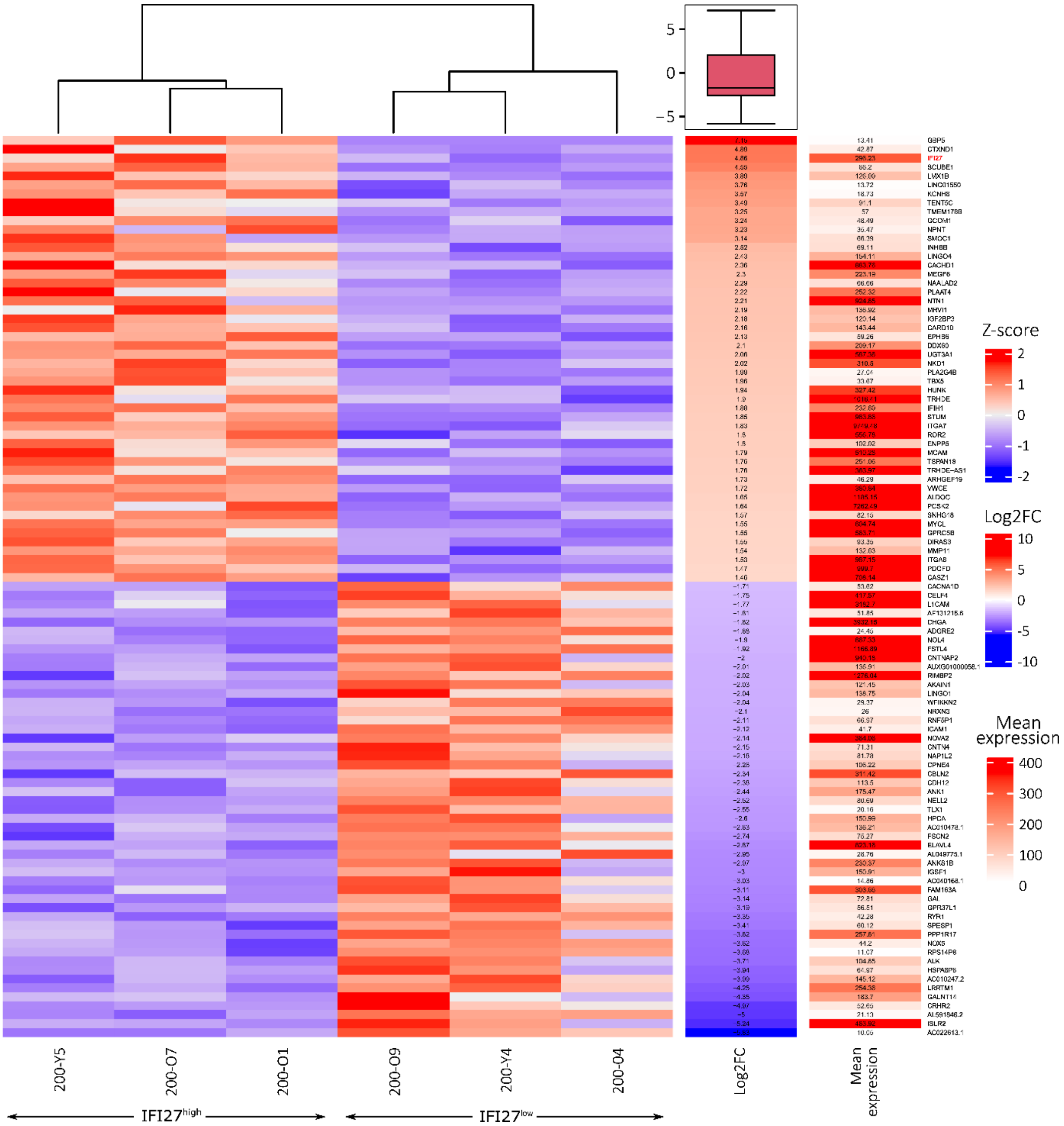

Figure S8 Genes associated with iNs derived from *IFI27*high and *IFI27*low expressing PLWH participants Heatmap showing the mean expression values of the top 50 up- and downregulated statistically significant (p-adj. < 0.05, log2fc > +/- 0.5) DEGs and the resulting log2 fold change between *IFI27*high and *IFI27*low expressing PLWH iNs based on our bulk-RNA analysis.

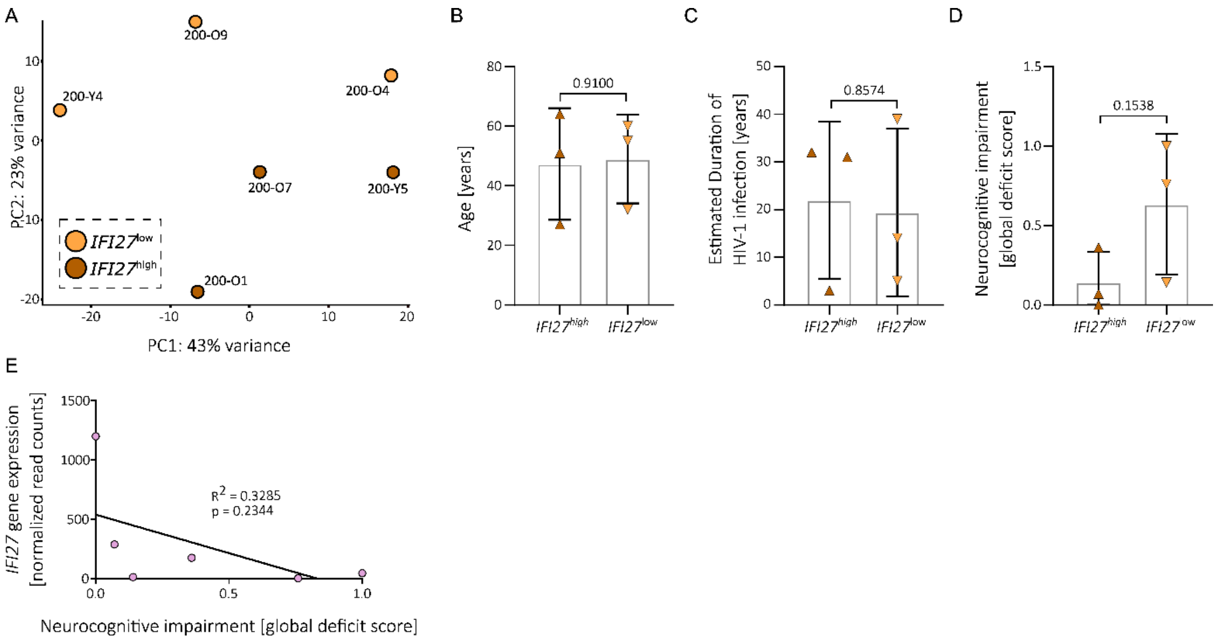

Figure S9 Biological parameters of the *IFI27*high and *IFI27*low expressing PLWH participants (**A**) PCA plot showing the calculated distance between the *IFI27*high and *IFI27*low expressing PLWH iNs samples. **(B-D)** Age **(B)**, estimated duration of HIV-1 infection **(C)**, and neurocognitive impairment measured as global deficit score **(D)** of PLWH (n = 6) divided into *IFI27*high vs. *IFI27*low expressing participants. Statistical significance tested with unpaired, two-tailed *t*-test. Data presented as individual data points with mean ± SD and p-value. **(E)** Correlation of *IFI27* gene expression levels with neurocognitive impairment in the PLWH study group (n =6). Data points are individual values illustrated together with a linear regression function.

References in supplements

1. Rippeth JD, Heaton RK, Carey CL, Marcotte TD, Moore DJ, Gonzalez R, et al. Methamphetamine dependence increases risk of neuropsychological impairment in HIV infected persons. *J Int Neuropsychol Soc.* 2004;10(1):1-14.
2. American Psychiatric Association., and American Psychiatric Association. Task Force on DSM-IV. *Diagnostic and statistical manual of mental disorders : DSM-IV-TR*. Washington, DC: American Psychiatric Association; 2000.
3. Kroenke K, Spitzer RL, and Williams JB. The PHQ-9: validity of a brief depression severity measure. *J Gen Intern Med.* 2001;16(9):606-13.
4. Evering TH, Applebaum A, La Mar M, Garmon D, Dorfman D, and Markowitz M. Rates of non-confounded HIV-associated neurocognitive disorders in men initiating combination antiretroviral therapy during primary infection. *AIDS.* 2016;30(2):203-10.
5. Carey CL, Woods SP, Gonzalez R, Conover E, Marcotte TD, Grant I, et al. Predictive validity of global deficit scores in detecting neuropsychological impairment in HIV infection. *J Clin Exp Neuropsychol.* 2004;26(3):307-19.
6. Heaton RK, Grant I, Butters N, White DA, Kirson D, Atkinson JH, et al. The HNRC 500--neuropsychology of HIV infection at different disease stages. HIV Neurobehavioral Research Center. *J Int Neuropsychol Soc.* 1995;1(3):231-51.
7. Mertens J, Herdy JR, Traxler L, Schafer ST, Schlachetzki JCM, Böhnke L, et al. Age-dependent instability of mature neuronal fate in induced neurons from Alzheimer's patients. *Cell Stem Cell.* 2021;28(9):1533-48.e6.

8. Zhou-Yang L, Eichhorner S, Karbacher L, Böhnke L, Traxler L, and Mertens J. Direct Conversion of Human Fibroblasts to Induced Neurons. *Methods Mol Biol*. 2021;2352:73-96.
9. Herdy J, Schafer S, Kim Y, Ansari Z, Zangwill D, Ku M, et al. Chemical modulation of transcriptionally enriched signaling pathways to optimize the conversion of fibroblasts into neurons. *Elife*. 2019;8.
10. Schindelin J, Arganda-Carreras I, Frise E, Kaynig V, Longair M, Pietzsch T, et al. Fiji: an open-source platform for biological-image analysis. *Nat Methods*. 2012;9(7):676-82.
11. Hao Y, Stuart T, Kowalski MH, Choudhary S, Hoffman P, Hartman A, et al. Dictionary learning for integrative, multimodal and scalable single-cell analysis. *Nat Biotechnol*. 2024;42(2):293-304.
12. Stuart T, Butler A, Hoffman P, Hafemeister C, Papalexi E, Mauck WM, et al. Comprehensive Integration of Single-Cell Data. *Cell*. 2019;177(7):1888-902.e21.
13. Satija R, Farrell JA, Gennert D, Schier AF, and Regev A. Spatial reconstruction of single-cell gene expression data. *Nat Biotechnol*. 2015;33(5):495-502.
14. Marsh SE. scCustomize: Custom Visualizations & Functions for Steamlined Analyses of Single Cell Sequencing. 2021. https://doi.org/10.5281/zenodo.5706430.
15. Schneider VA, Graves-Lindsay T, Howe K, Bouk N, Chen HC, Kitts PA, et al. Evaluation of GRCh38 and de novo haploid genome assemblies demonstrates the enduring quality of the reference assembly. *Genome Res*. 2017;27(5):849-64.
16. Dobin A, Davis CA, Schlesinger F, Drenkow J, Zaleski C, Jha S, et al. STAR: ultrafast universal RNA-seq aligner. *Bioinformatics*. 2013;29(1):15-21.
17. Anders S, Pyl PT, and Huber W. HTSeq--a Python framework to work with high-throughput sequencing data. *Bioinformatics*. 2015;31(2):166-9.
18. Love MI, Huber W, and Anders S. Moderated estimation of fold change and dispersion for RNA-seq data with DESeq2. *Genome Biol*. 2014;15(12):550.
19. Zhu A, Ibrahim JG, and Love MI. Heavy-tailed prior distributions for sequence count data: removing the noise and preserving large differences. *Bioinformatics*. 2019;35(12):2084-92.
20. Anders S, and Huber W. Differential expression analysis for sequence count data. *Genome Biol*. 2010;11(10):R106.
21. Chen EY, Tan CM, Kou Y, Duan Q, Wang Z, Meirelles GV, et al. Enrichr: interactive and collaborative HTML5 gene list enrichment analysis tool. *BMC Bioinformatics*. 2013;14:128.
22. Kuleshov MV, Jones MR, Rouillard AD, Fernandez NF, Duan Q, Wang Z, et al. Enrichr: a comprehensive gene set enrichment analysis web server 2016 update. *Nucleic Acids Res*. 2016;44(W1):W90-7.
23. Xie Z, Bailey A, Kuleshov MV, Clarke DJB, Evangelista JE, Jenkins SL, et al. Gene Set Knowledge Discovery with Enrichr. *Curr Protoc*. 2021;1(3):e90.
24. Rhee HW, Zou P, Udeshi ND, Martell JD, Mootha VK, Carr SA, et al. Proteomic mapping of mitochondria in living cells via spatially restricted enzymatic tagging. *Science*. 2013;339(6125):1328-31.
25. Subramanian A, Tamayo P, Mootha VK, Mukherjee S, Ebert BL, Gillette MA, et al. Gene set enrichment analysis: a knowledge-based approach for interpreting genome-wide expression profiles. *Proc Natl Acad Sci U S A*. 2005;102(43):15545-50.
26. Ashburner M, Ball CA, Blake JA, Botstein D, Butler H, Cherry JM, et al. Gene ontology: tool for the unification of biology. The Gene Ontology Consortium. *Nat Genet*. 2000;25(1):25-9.
27. Aleksander SA, Balhoff J, Carbon S, Cherry JM, Drabkin HJ, Ebert D, et al. The Gene Ontology knowledgebase in 2023. *Genetics*. 2023;224(1).
28. Grissa D, Junge A, Oprea TI, and Jensen LJ. Diseases 2.0: a weekly updated database of disease-gene associations from text mining and data integration. *Database (Oxford)*. 2022;2022.

29. Koopmans F, van Nierop P, Andres-Alonso M, Byrnes A, Cijsouw T, Coba MP, et al. SynGO: An Evidence-Based, Expert-Curated Knowledge Base for the Synapse. *Neuron*. 2019;103(2):217-34.e4.
30. Wu G, and Haw R. Functional Interaction Network Construction and Analysis for Disease Discovery. *Methods Mol Biol*. 2017;1558:235-53.
31. Milacic M, Beavers D, Conley P, Gong C, Gillespie M, Griss J, et al. The Reactome Pathway Knowledgebase 2024. *Nucleic Acids Res*. 2024;52(D1):D672-D8.
32. Piñero J, Ramírez-Anguita JM, Saüch-Pitarch J, Ronzano F, Centeno E, Sanz F, et al. The DisGeNET knowledge platform for disease genomics: 2019 update. *Nucleic Acids Res*. 2020;48(D1):D845-D55.
33. Liberzon A, Birger C, Thorvaldsdóttir H, Ghandi M, Mesirov JP, and Tamayo P. The Molecular Signatures Database (MSigDB) hallmark gene set collection. *Cell Syst*. 2015;1(6):417-25.
34. Patro R, Duggal G, Love MI, Irizarry RA, and Kingsford C. Salmon provides fast and bias-aware quantification of transcript expression. *Nat Methods*. 2017;14(4):417-9.
35. Mertens J, Paquola ACM, Ku M, Hatch E, Böhnke L, Ladjevardi S, et al. Directly Reprogrammed Human Neurons Retain Aging-Associated Transcriptomic Signatures and Reveal Age-Related Nucleocytoplasmic Defects. *Cell Stem Cell*. 2015;17(6):705-18.
36. Ren X, and Kuan PF. RNAAgeCalc: A multi-tissue transcriptional age calculator. *PLoS One*. 2020;15(8):e0237006.
37. Meyer DH, and Schumacher B. Aging clocks based on accumulating stochastic variation. *Nat Aging*. 2024;4(6):871-85.
38. Robinson MD, McCarthy DJ, and Smyth GK. edgeR: a Bioconductor package for differential expression analysis of digital gene expression data. *Bioinformatics*. 2010;26(1):139-40.
39. Meyer DH, and Schumacher B. BiT age: A transcriptome-based aging clock near the theoretical limit of accuracy. *Aging Cell*. 2021;20(3):e13320.
40. Seabold S, and Perktold J. Proceedings of the 9th Python in Science Conference: SciPy; 2010:92-6.
41. Del Toro N, Shrivastava A, Ragueneau E, Meldal B, Combe C, Barrera E, et al. The IntAct database: efficient access to fine-grained molecular interaction data. *Nucleic Acids Res*. 2022;50(D1):D648-D53.
42. Luck K, Kim DK, Lambourne L, Spirohn K, Begg BE, Bian W, et al. A reference map of the human binary protein interactome. *Nature*. 2020;580(7803):402-8.
43. Blazkova J, Boughorbel S, Presnell S, Quinn C, and Chaussabel D. A curated transcriptome dataset collection to investigate the immunobiology of HIV infection. *F1000Res*. 2016;5:327.
44. Harrison PW, Amode MR, Austine-Orimoloye O, Azov AG, Barba M, Barnes I, et al. Ensembl 2024. *Nucleic Acids Res*. 2024;52(D1):D891-D9.
